# Pneumococcal Extracellular Vesicles Modulate Host Immunity

**DOI:** 10.1128/mBio.01657-21

**Published:** 2021-07-13

**Authors:** Saigopalakrishna S. Yerneni, Sarah Werner, Juliana H. Azambuja, Nils Ludwig, Rory Eutsey, Surya D. Aggarwal, Peter C. Lucas, Nathanael Bailey, Theresa L. Whiteside, Phil G. Campbell, N. Luisa Hiller

**Affiliations:** a Department of Biomedical Engineering, Carnegie Mellon University, Pittsburgh, Pennsylvania, USA; b Department of Biological Sciences, Carnegie Mellon University, Pittsburgh, Pennsylvania, USA; c UPMC Hillman Cancer Center, University of Pittsburgh, Pittsburgh, Pennsylvania, USA; d Department of Pathology, University of Pittsburgh School of Medicine, Pittsburgh, Pennsylvania, USA; e Department of Oral and Maxillofacial Surgery, University Hospital Regensburg, Regensburg, Germany; f Departments of Immunology and Otolaryngology, Pittsburgh, Pennsylvania, USA; g Engineering Research Accelerator, Carnegie Mellon University, Pittsburgh, Pennsylvania, USA; University of Mississippi Medical Center

**Keywords:** Gram-positive bacteria, *Streptococcus pneumoniae*, extracellular vesicles, EVs, macrophage signaling, alternative activation pathway, host-pathogen interactions, immune response, pathogenesis

## Abstract

Extracellular vesicles (EVs) have recently garnered attention for their participation in host-microbe interactions in pneumococcal infections. However, the effect of EVs on the host immune system remain poorly understood. Our studies focus on EVs produced by *Streptococcus pneumoniae* (pEVs), and reveal that pEVs are internalized by macrophages, T cells, and epithelial cells. *In vitro*, pEVs induce NF-κB activation in a dosage-dependent manner and polarize human macrophages to an alternative (M2) phenotype. In addition, pEV pretreatment conditions macrophages to increase bacteria uptake and such macrophages may act as a reservoir for pneumococcal cells by increasing survival of the phagocytosed bacteria. When administered systemically in mice, pEVs induce cytokine release; when immobilized locally, they recruit lymphocytes and macrophages. Taken together, pEVs emerge as critical contributors to inflammatory responses and tissue damage in mammalian hosts.

## INTRODUCTION

Extracellular vesicles (EVs), naturally occurring lipid bilayer vesicles that contain extracellular, membrane and cytosolic signaling agents, are produced by all cells (1–3). EVs serve as cell-to-cell mailing systems that enable highly effective cellular communication between cells within and across eukaryotic and prokaryotic domains. To accomplish this function, signaling cargos are selectively packaged into and onto EVs, which are then secreted into the extracellular milieu where this cargo is delivered to target cells eliciting biological responses.

Microbial EVs have long been studied in Gram-negative bacteria. In these bacteria, EVs bud from the outer cell membrane and thus are referred to as outer membrane vesicles (OMVs) (4). OMVs promote collaborative interactions in the bacterial environment (5), delivering a broad variety of cargo, including toxins, nutrients (6, 7), enzymes (8, 9), and nucleic acids (10, 11). These activities can influence sensitivity of bacteria to antibiotics and alter the ability to develop biofilms. OMVs also have beneficial autocrine effects on the cells that secrete them (3). Under stress conditions, OMVs loaded with misfolded proteins or toxic compounds bud from the cell, relieving the cell of these harmful components (12). Finally, they can modify immune cells in a manner that either promotes or inhibits host defenses (13). Overall, OMVs are EVs of Gram-negative origin that interact with bacterial and host cells and manipulate their biological processes.

While Gram-negative EVs were first reported in 1966 (14), Gram-positive bacteria were assumed to be incapable of EV release due to the thickness of their cell wall until recently (2). Recent studies in Staphylococcus aureus (15–17), Bacillus anthracis (18), group A Streptococcus (19), and Streptococcus pneumoniae (20–22) strongly support the production of EVs by Gram-positive bacteria (23–25). Gram-positive EVs are now thought to contribute to biofilm development and antibiotic resistance (26, 27) and deliver phage and toxin receptors, sensitizing cells to attack by phages and other bacteria (28–30). Thus, like OMVs, EVs from Gram-positive cells interact with bacterial and host cells to manipulate various biological processes.

This study focuses on Streptococcus pneumoniae (pneumococcus) EVs, which we refer to as pEVs (pneumococcal EVs). Relative to the bacterial source, pEVs are enriched in lipoproteins and short-chain fatty acids and carry a multitude of surface proteins and virulence determinants (20, 22). Some examples of these virulence determinants include the surface proteins PspA and PspC and the pneumolysin toxin (20, 22). Further, these vesicles bud from the bacterial surface in the outside-out orientation, such that molecules on pEVs maintain the same orientation as that in bacterial cells (20). Inoculation of mice with pEVs induces antibody production and provides protection against pneumococcal infection, suggesting that pEVs have potential as vaccines (22, 31). In addition, pEVs isolated from serotype 4 pneumococcus sequester components of the complement system and may serve as a decoy against innate immune complement-mediated killing of pneumococci (20). pEVs are also internalized by dendritic cells, where they induce proinflammatory cytokine responses (20), and by lung epithelial cells, keratinocytes, and monocytes, which tolerate high concentration of EVs without compromising host cell viability (32). Finally, pEV-associated DNase TatD may enable the evasion of pneumococci from neutrophil extracellular traps, which are DNA-based webs released by neutrophils to immobilize pathogens (21). Thus, pEVs interact with host cells and influence complement-mediated killing and adaptive immunity.

Pneumococcal colonization precedes dissemination and disease. Interactions between the bacteria and the host innate immune response are critical to the outcome of infection. We set out to understand how pEVs influence innate immunity and to define the mechanism(s) underpinning these EV-mediated effects. Our *in vitro* studies showed that pEVs were internalized by macrophages and induced nuclear factor-κB (NF-κB) activation in a dose-dependent manner. In mice, pEVs induced potent immunomodulatory effects. Systemic delivery of pEVs triggered a dramatic induction of cytokines in plasma and induced a germinal center reaction in the spleen, where macrophages engulf apoptotic lymphocyte debris (tingible body macrophages). In addition, with localized administration, site-retained pEVs resulted in immune cell recruitment to the delivery site, suggesting that pEVs promote migration of immune cells to the site of inoculation. Comparison of bacteria and pEVs exposure to primary human macrophages revealed that pEVs, but not bacteria, stimulated an alternative polarization (M2 phenotype). Finally, pEV pretreatment increased the phagocytic capacity of primary human macrophages but made them less efficient in killing the phagocytosed bacteria. These data suggest that pEVs play a critical role in pneumococcus-host interactions and demonstrate the ability of pEVs to trigger immune responses and redirect macrophage activation.

## RESULTS

### Isolation and characterization of pEVs from *Streptococcus pneumoniae*.

**FIG 1:**
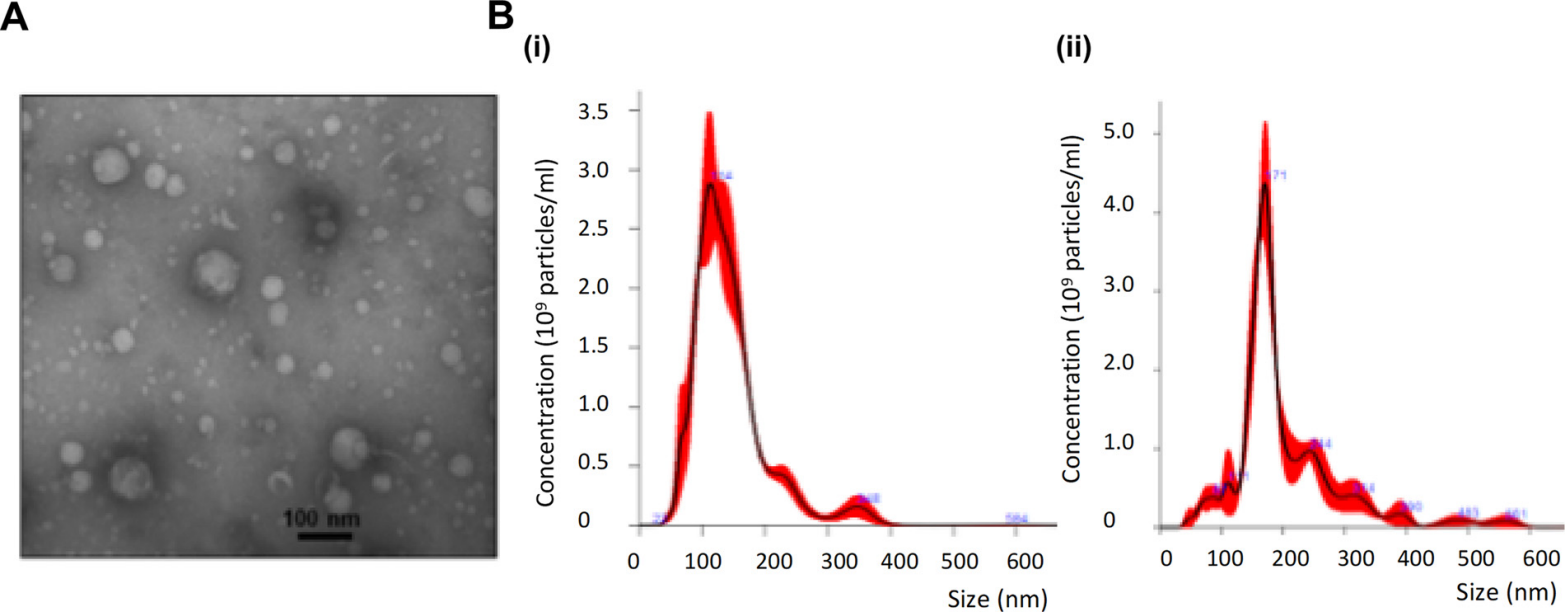
Viable pneumococci produce EVs. (A) Representative transmission electron micrograph image of pEVs purified by SEC from strain R6. (B) Representative output from nanoparticle tracking analyses of pEVs, displaying size distribution and concentration. Bacterial cells used for purification were grown (i) in rich media or (ii) in rich media supplemented with 2% choline chloride to inhibit autolysis (*n* = 2).

We employed size exclusion chromatography (SEC) to isolate pEVs from bacteria conditioned culture broth. R6 strain, a nonencapsulated derivative of strain D39, was selected as a model strain since previous work comparing pEV production between R6 and encapsulated strains reported decreased yields in encapsulated strains, presumably due to the physical barrier of the capsule (22). pEVs were harvested from bacterial cultures grown to late log phase; they were visualized by transmission electron microscopy (Fig. 1A; see also Fig. S1A in the supplemental material) and quantified and enumerated by NanoSight (Fig. 1Bi). The pEVs ranged from 27 to 400 nm in diameter, the mode size was 127 nm. A representative SEC purification run produced 5 × 10^9^ vesicles per 500 ml of late-exponential culture, which corresponds to a final protein yield of 45 ± 6.5 μg for 1 ml of purified EVs.

10.1128/mBio.01657-21.1 FIG S1 Pneumococcal cell with attached pEV. Representative electron microscopy image of pneumococcus with attached pEV (red arrow). Download FIG S1, TIF file, 2.6 MB. Copyright © 2021 Yerneni et al. 2021 Yerneni et al. https://creativecommons.org/licenses/by/4.0/ This content is distributed under the terms of the Creative Commons Attribution 4.0 International license.

To determine whether bacterial cell death contributed to pEV production, we assessed the number of dead cells in the producing cultures and quantified pEV production when autolysis was inhibited. Confocal microscopy of bacterial cultures stained with live/dead stain revealed that >95% of cells were viable at the time of EV collection and purification. Pneumococcal autolysis can be blocked by growing cells in choline chloride since lysis requires the attachment of choline-binding proteins to the cell surface (33–36). The yield and size of pEVs did not substantially vary in a condition upon blocking autolysis: the mode size was 159 nm, and a representative SEC purification run produced 4.7 × 10^9^ vesicles per 500 ml of culture (Fig. 1Bii). We conclude that viable pneumococcal cells produce pEVs during exponential growth.

### pEVs are internalized by host cells.

**FIG 2:**
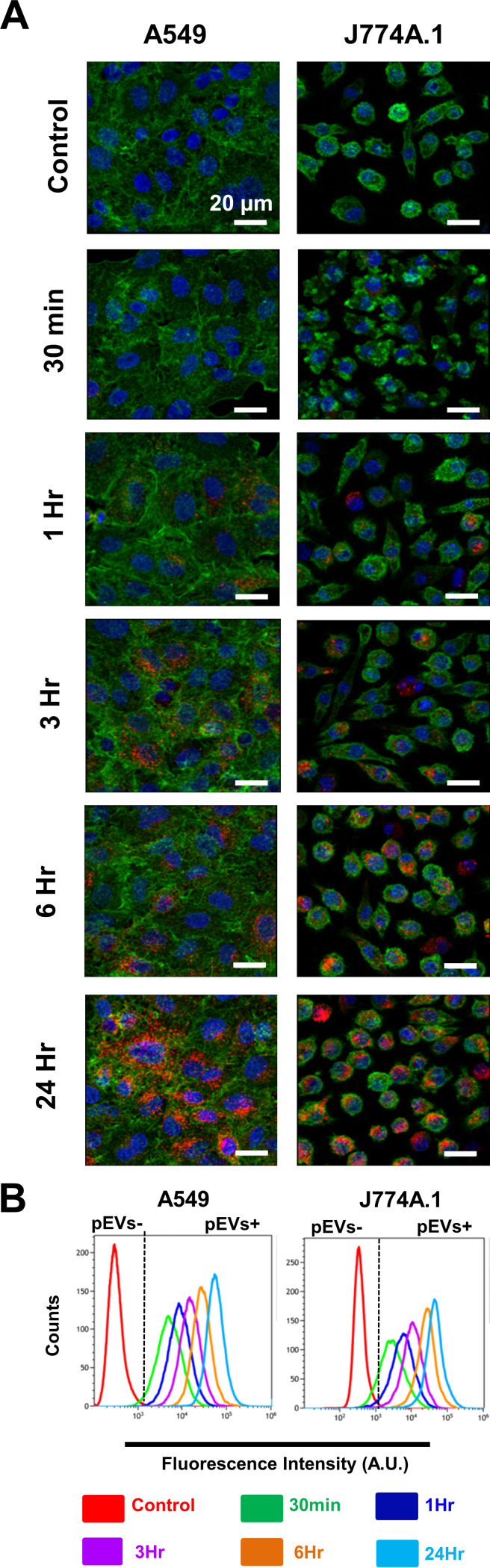
pEVs are internalized by host cells. (A) Representative confocal images of A549 lung epithelial cells (left panels) and murine macrophages (J774A.1; right panels) exposed to pEVs for the indicated times. Red, DiD-stained pEVs; green, F-actin; blue, DAPI nuclear stain. (B) Representative flow cytometry measurements of the amounts of DiD detected in cells before the addition of pEVs (control) and at five time points after the addition of 20 μg/ml of pEVs (*n* = 2). The red line demonstrates the fluorescence intensity of cells without any pEVs. All cells exhibited pEV internalization by 24 h.

During human infections, the pneumococcus encounters both epithelial cells and immune cells. pEVs have previously been shown to be internalized by lung epithelial cells A549 and monocyte-derived dendritic cells (20), and we were able to validate these findings with regard to A549. Further, we investigated whether pEVs were internalized into mouse macrophages (J774A.1) and primary CD4^+^/CD8^+^ T cells (Fig. 2; see also Fig. S2). To this end, pEVs were stained with Vibrant DiD lipophilic dye (ThermoFisher), exposed to host cells for 30 min, 1 h, 3 h, 6 h, or 24 h, and analyzed by confocal microscopy and flow cytometry. Prior to analysis, the cells were treated with acid rinsing to wash-off any cell membrane associated pEVs. The internalization of pEVs occurred in a time-dependent manner (Fig. 2; see also Fig. S2). The extent of internalization is demonstrated by a three-dimensional reconstruction where pEVs are detected within an A549 cell (see Video S1). All four cell types internalized pEVs by 24 h, suggesting that the bacterial protein cargo constituents associated with pEVs are delivered into host cells.

10.1128/mBio.01657-21.2 FIG S2 pEVs are internalized by T cells. Representative flow cytometry measurements of the amount of DiD label detected in CD4^+^ and CD8^+^ T cells before addition of pEVs (control) and at five time points after addition of 20 μg/ml of pEVs (*n* = 3) are presented. The red line demonstrates the fluorescence intensity of cells without any pEVs. All cells exhibited pEV internalization by 24 h. Download FIG S2, TIF file, 1.2 MB. Copyright © 2021 Yerneni et al. 2021 Yerneni et al. https://creativecommons.org/licenses/by/4.0/ This content is distributed under the terms of the Creative Commons Attribution 4.0 International license.

### pEVs induce NF-κB signaling in macrophages.

**FIG 3:**
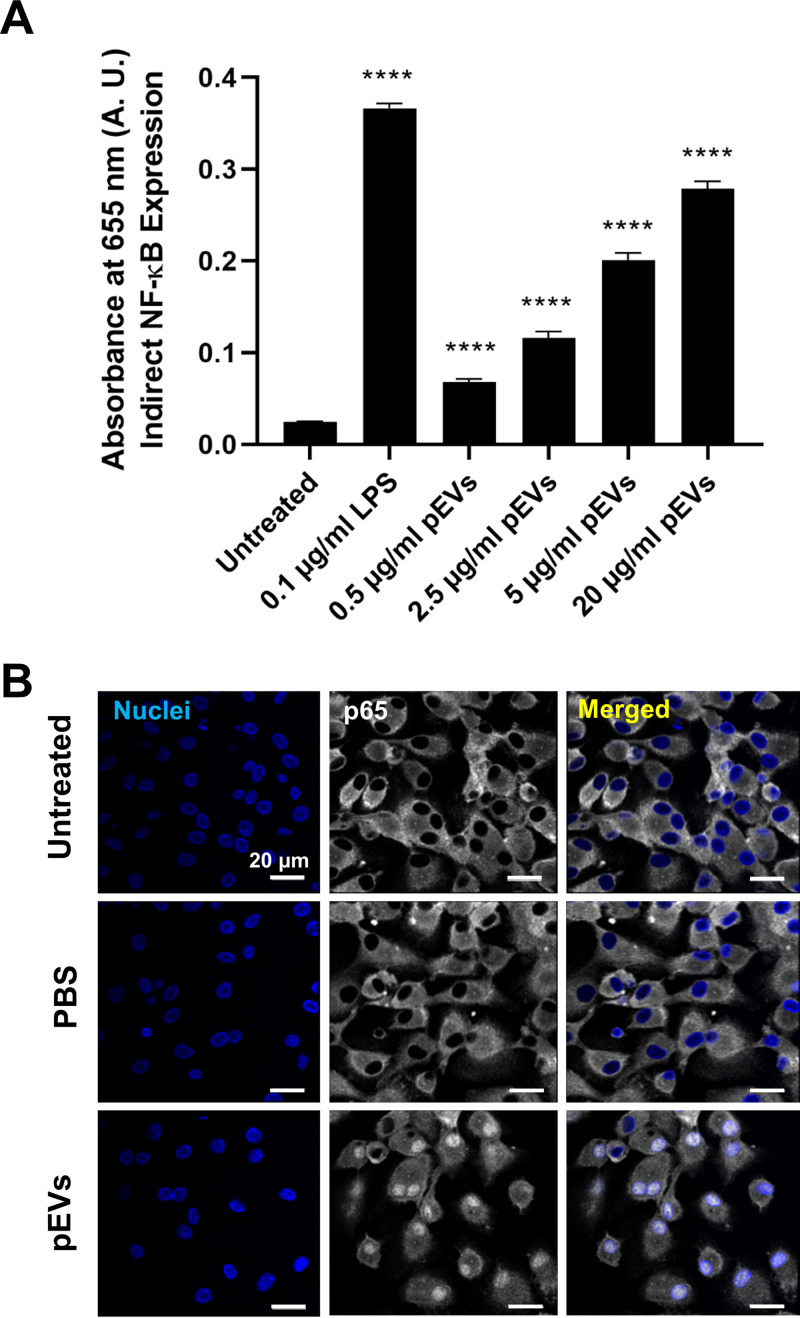
pEVs induce NK-κB signaling in macrophages. (A) Graph of NF-κB production in response to various concentrations of pEVs contrasted to negative (no pEVs; untreated) and positive (0.1 μg/ml LPS) controls. Measurements from a murine macrophage reporter cell line where secreted fetal alkaline phosphatase is under the control of NF-κB. The supernatant of the cell line was assayed for reporter activity 24 h postexposure to pEVs representing cumulative reporter release and overall NF-κB activation (*n* = 2). (B) Representative confocal images of NF-κB activation in primary human macrophages, 30 min after exposure to pEVs (20 μg/ml) or control groups (*n* = 2). Blue, DAPI nuclear stain; white, p65.

Macrophages are critical components of innate immunity and among the first cells to respond to incoming bacteria. Naturally, we were interested in investing the role of pEVs in inducing signaling responses in macrophages and, since NF-κB is a master regulator of inflammatory responses, we measured NF-κB signaling in macrophages after exposure to pEVs (37). We used a murine macrophage reporter cell line where secreted alkaline phosphatase is under the control of NF-κB. Doses of 500 ng/ml to 20 μg/ml of pEVs induced NF-κB activation in a dose-dependent manner (Fig. 3A). Further, we studied NF-κB induction in primary human macrophages by staining for p65, which translocates from cytoplasm to nucleus upon NF-κB activation. The pEVs, but not the controls (no treatment and phosphate-buffered saline [PBS] treatment), induced NF-κB, as determined by p65 translocation to the nucleus within 30 min (Fig. 3B). Overall, pEVs induced robust NF-κB signaling in macrophages in a dose-dependent manner.

### Systemic delivery of pEVs promotes broad host responses.

The interaction between pEVs and the innate immune response has not been explored *in vivo*. We therefore investigated whether pEVs induce immune responses in a mammalian host by performing intravenous injections of pEVs in C57BL/6J mice. Mice were injected with pEVs or PBS as a control. The PBS control consisted of bacterial media eluted off a Sepharose 2B column with PBS and thus treated in the same exact manner as the pEV fractions. At 24 h postinoculation, mice were euthanized and analyzed for changes in the spleen and peripheral blood mononuclear cells (PBMCs).

**FIG 4:**
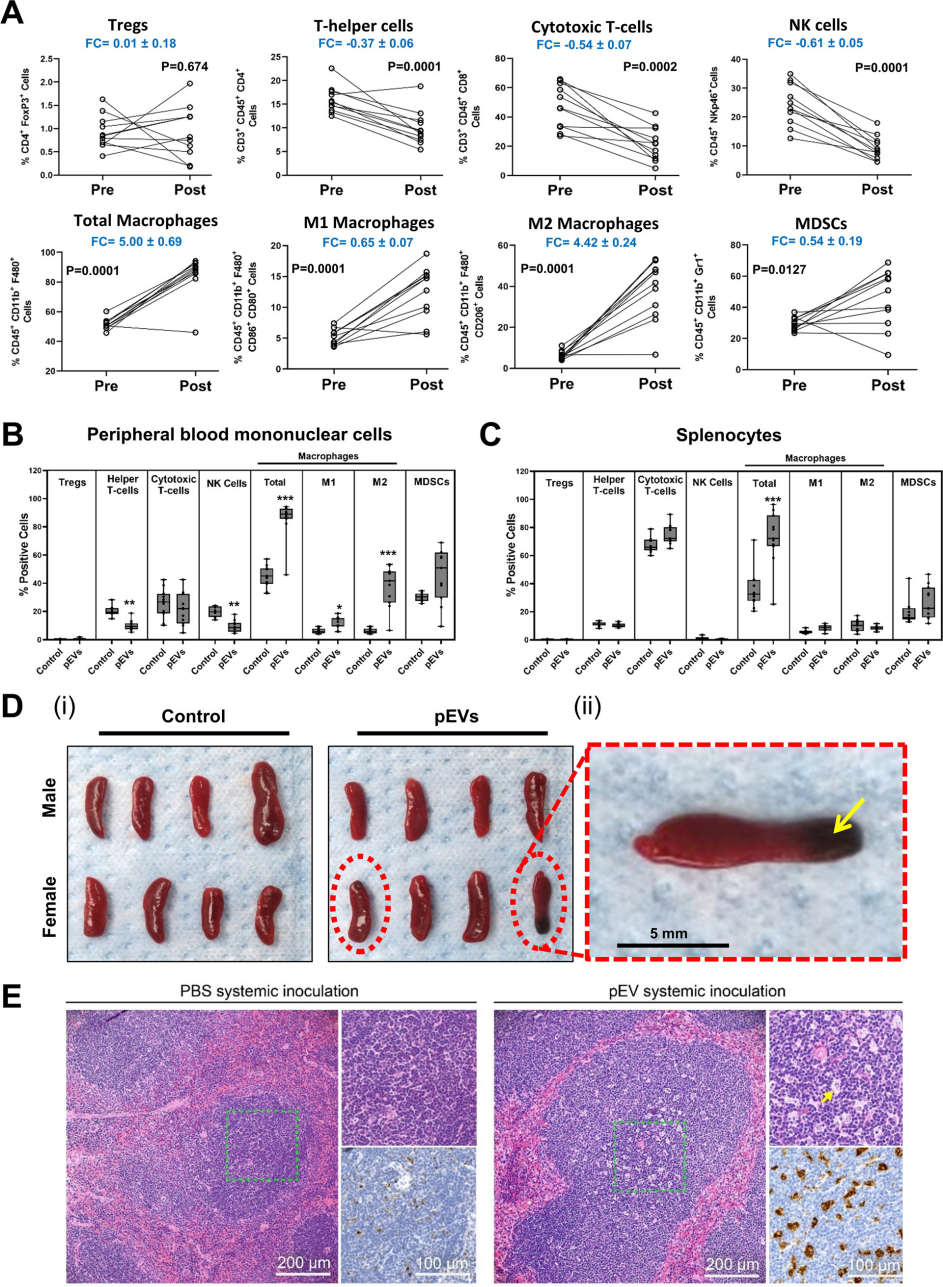
Systemic inoculation of pEVs in mice induces immune changes. (A and B) Cell markers were used to assess changes in PBMC and splenocyte populations. The cell types assessed were as follows: regulatory T cells (CD4^+^ and FoxP3^+^), helper T cells (CD3^+^, CD45^+^, and CD4^+^), cytotoxic T cells (CD3^+^, CD45^+^, and CD8^+^), all macrophages (CD45^+^, CD11b^+^, and F480^+^), M1 macrophages (CD45^+^, CD11b^+^, F480^+^, CD86^+^, and CD80^+^), M2 macrophages (CD45^+^, CD11b^+^, F480^+^, and CD206^+^), natural killer cells (CD45^+^ and NKp46^+^), and myeloid-derived suppressor cells (CD45^+^, CD11b^+^, and Gr1^+^). (A) Mice were inoculated intravenously with pEVs purified from wild-type bacteria (0.1 ml with 15 μg of pEVs) and analyzed for relative changes in PBMC populations preinoculation and 24 h postinoculation (*n* = 11 animals per cohort over two independent experiments). FC, fold change. (B) Postinoculation PBMCs identified as percent positive populations, comparing mice inoculated with pEVs to PBS control group. (C) Postinoculation splenocyte identified as percent positive populations, comparing pEV inoculations to PBS control group. (B and C) Box-and-whisker plots show median, range 25 to 75%, and min-max values and *n* = 11 for pEV and for control groups. Data were collected over two independent experiments. *, *P* ≤ 0.05; **, *P* ≤ 0.01 (versus control group). (D) Gross anatomy of excised spleens from the first experimental set of eight animals. (i) Red dashed ellipsoids highlight macroscopic legions, (ii) whereas a yellow arrow denotes a region with necrosis. (E) Representative histological images of spleens from mice treated with PBS (left panels) or pEVs (right panels). Within each group, the left image is stained with H&E, whereas the right upper panel represents a magnified region from the green dashed rectangle of the corresponding left panel. The yellow arrow identifies tingible body macrophages. The right lower panel is a representative image of immunostaining with CD68, a general macrophage marker.

The intravenous administration of pEVs resulted in systemic changes. The distinct cell types within PBMCs were quantified by flow cytometry based on a selected panel of 12 immune markers (Fig. 4A and B). A comparison of each animal pre- and post-pEV treatment revealed substantial changes in the PBMC composition (Fig. 4A). Notably, treatment with pEVs induced a 5-fold increase in the total macrophage population. We observed a 4.4-fold (4.42 ± 0.24) increase in M2 macrophages and a 0.6-fold (0.65 ± 0.07) increase in M1 macrophages. Also significant was the 0.6-fold (0.61 ± 0.05) decrease in NK cells, as well as a mild decrease in both T-helper and cytotoxic T cells. In accordance with this, the same effects were observed when we compared posttreatment animals treated with pEVs versus PBS controls (Fig. 4B).

Pneumococcal infection also induces changes in the spleen. Macroscopic analysis of the spleens revealed black patches in three spleens (out of eleven) from mice exposed to pEVs, typical of regions of necrosis (Fig. 4D). Quantification of the immune cell populations in the spleen revealed that animals treated with pEVs displayed a marked increase in the relative percentages of splenic macrophages compared to the PBS controls (Fig. 4C). Moreover, microscopic analysis of splenic tissue stained with hematoxylin and eosin (H&E) revealed that pEV administration induced white pulp expansion due to increased germinal center formation. Furthermore, the germinal centers displayed numerous tingible body macrophages; these are macrophages that are in the process of engulfing pieces of apoptotic lymphocyte debris that result from the simultaneous proliferation and apoptosis that occurs in the setting of germinal center reaction (38, 39). This was supported by staining for macrophages with anti-CD68, which confirmed a qualitative increase in macrophages in spleens from pEV-treated animals relative to PBS controls (Fig. 4E). Overall, we conclude that systemic delivery of pEVs triggers a strong immune response in the blood and in the spleen. In the spleen, diverse lymphocyte populations expand and undergo cell death. In the blood, we measured an increase in macrophages consistent with induction of the innate immune response.

### Biological functions of pEVs are partially mediated by lipoproteins.

**FIG 5:**
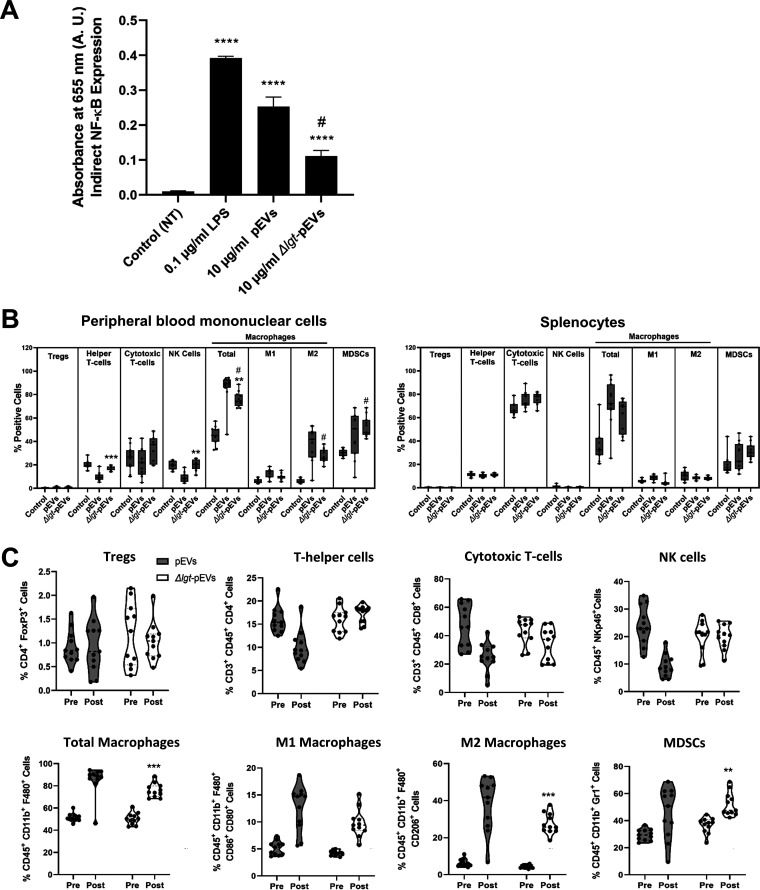
Lipoproteins associated with pEVs contribute to macrophage signaling and alter PBMC landscape. (A) NF-κB production in murine macrophage reporter cell line (RAW-Blue) is reduced for *Δlgt-*pEVs compared to pEVs. Bars represent the means ± the standard errors of the mean (SEM) (*n* = 3). ****, *P* ≤ 0.0001 (versus control group); #, *P* ≤ 0.0001 (versus pEV group). (B) Cell markers were used to determine the nature and the relative quantification of PBMCs and splenocytes. The cell types assessed were defined as follows: regulatory T cells (CD4^+^ and FoxP3^+^), helper T cells (CD3^+^, CD45^+^, and CD4^+^), cytotoxic T cells (CD3^+^, CD45^+^, and CD8^+^), all macrophages (CD45^+^, CD11b^+^, and F480^+^), M1 macrophages (CD45^+^, CD11b^+^, F480^+^, CD86^+^, and CD80^+^), M2 macrophages (CD45^+^, CD11b^+^, F480^+^, and CD206^+^), natural killer cells (CD45^+^ and NKp46^+^), and myeloid-derived suppressor cells (CD45^+^, CD11b^+^, and Gr1^+^). Postinoculation PBMCs are identified as percent positive populations, comparing mice inoculated with pEVs from the wild type, pEVs from the Δ*lgt* strain, and the PBS control group. Asterisks (*) represent statistical significance relative to pEV from wild-type cells, and number symbols (#) represent statistical significance relative to controls. Box-and-whisker plots show median, range 25 to 75%, and min-max values (*n* = 11 per cohort). (C) Violin plots (*n* = 11 per cohort) showing relative percentages of PBMC pre- and posttreatment with wild-type pEVs (gray) and *Δlgt*-pEVs (white). *, *P* ≤ 0.05; **, *P* ≤ 0.01; ***, *P* ≤ 0.001; ****, *P* ≤ 0.0001.

Mass spectroscopy studies have demonstrated that pEVs are enriched in lipoproteins (22). Further, immunological studies revealed that lipoproteins are critical for TLR2 signaling on macrophages and consequently contribute to the induction of multiple proinflammatory cytokines (40). Thus, we hypothesized that lipoproteins contribute to pEV-mediated macrophage signaling. To test this hypothesis, we utilized pEVs from a deletion mutant of the lipoprotein diacylglyceryl transferase (gene *lgt*), since deletion of this protein leads to loss of surface lipoproteins (41). The extent of NF-κB signaling was significantly lower in response to pEVs from *Δlgt* cells compared to induction by pEVs from wild-type cells (Fig. 5A), suggesting that lipoproteins contribute to pEV-mediated NF-κB induction in macrophages.

The influence of bacterial lipoproteins on pEV function was also observed in the systemic administration of pEVs into mice. We compared the responses to systemic administration of pEVs purified from wild-type cells to those of pEVs purified from *Δlgt* cells. While wild-type pEVs contributed to a decrease in NK cells and helper T cells in PBMCs (Fig. 4B), this decrease was not observed with pEVs from the *Δlgt* strain, indicating the contribution of lipoproteins to this phenotype (Fig. 5B). In contrast, a role for lipoproteins in influencing the pEV-mediated increase in macrophage populations in either blood or spleen was minimal, because pEVs from the *Δlgt* strain affected macrophage populations similar to the wild-type strain (Fig. 5B and C).

**FIG 6:**
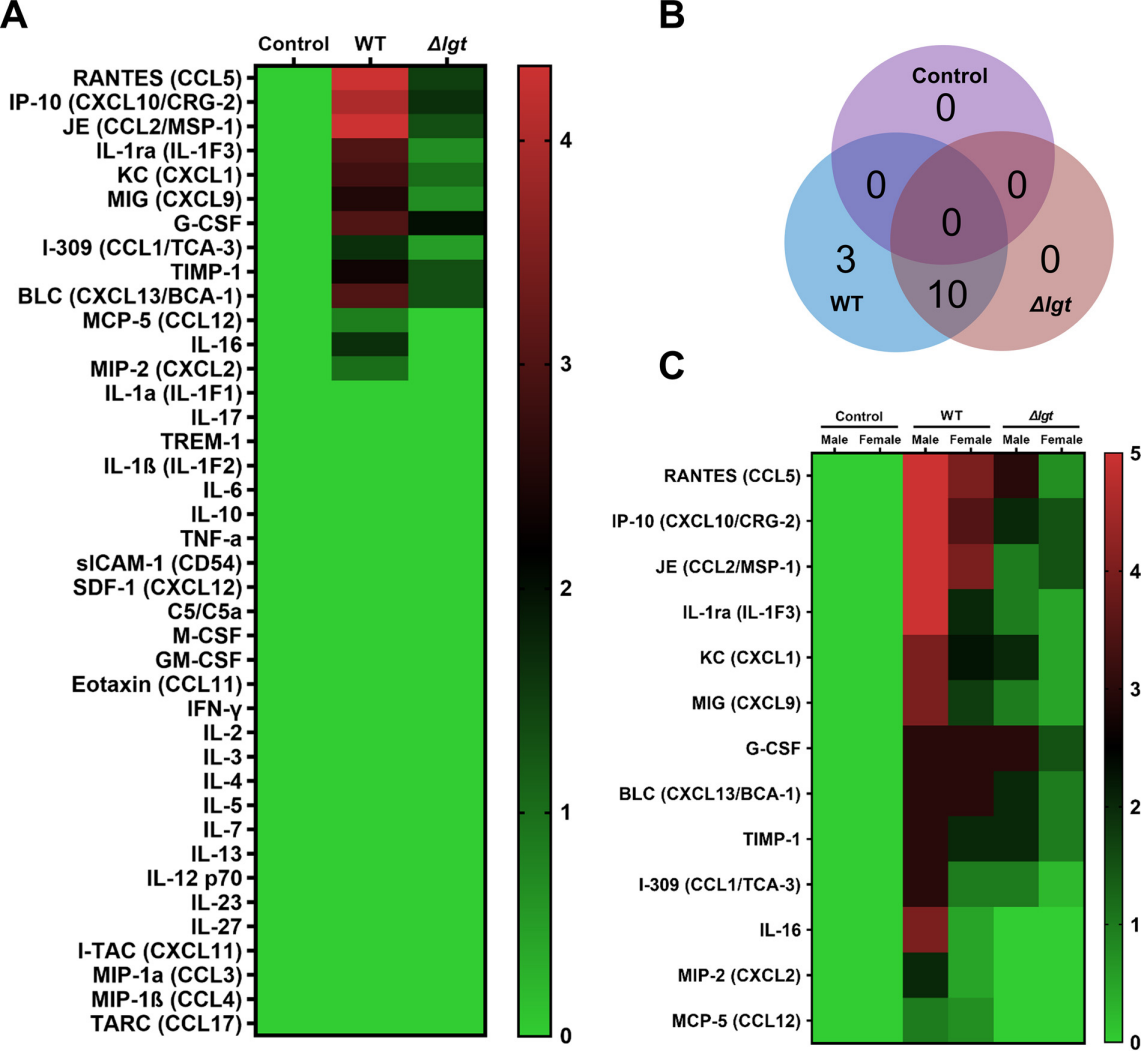
Systemic inoculation of pEVs triggers induction of immune markers in mice. (A) Heat map of 40 immune markers in mouse plasma. Mice were inoculated intravascularly with PBS control, pEVs (15 μg in 100 μl), or *Δlgt-*pEVs (15 μg in 100 μl). Experiments were performed in triplicate, each from a set of pooled plasma from three mice. (B) Venn diagram of data highlighting the number of cytokines where the concentration was changed between pre- and postinoculation (heat map value 1 to 5) in each group (independent of mice sex). (C) Heat map of 13 cytokines upregulated in plasma, displayed considering sex as a biological variable where male and female mice are plotted separately. Data from the females represent the average of two cohorts, while data from males represent one cohort. Mice were inoculated intravascularly with PBS, pEVs, or *Δlgt-*pEVs. The ELISA blots are presented in Fig. S3.

Finally, we tested whether pEVs, like bacterial cells, can trigger cytokine secretion in the blood and established the contribution of lipoproteins to this process. To this end, an enzyme-linked immunosorbent assay (ELISA) array with 40 immune markers (cytokines and growth factors associated with immune responses) was employed. Three sets of plasma samples were analyzed for fold change independently, each one corresponding to pooled plasma from three sex-matched mice (two sets from females and one set from males). Thirteen markers displayed an increase after inoculation with pEVs in at least one of these sets (Fig. 6; see also Fig. S3). The most pronounced and consistent changes were observed for RANTES (CCL5), JE (MSP-1/CCL2), and IP-10 (CXCL10). More than half of the induced markers include chemokines of the CXCL and CCL families, suggesting that pEVs contribute to immune cell recruitment. Experiments with pEVs from the *Δlgt* strain established that these molecules alter the number and extent of cytokine secretion in plasma. Compared to pEVs from wild-type cells, the pEVs from the *Δlgt* strain led to induction of a smaller number of cytokines, and for those induced, many displayed decreased induction levels (Fig. 6; see also Fig. S3). Overall, these data suggest that pEVs promote cytokine signaling and that lipoproteins carried by pEVs contribute to this systemic immunomodulatory effect.

10.1128/mBio.01657-21.3 FIG S3 Detection of cytokines in mice inoculated systemically with pEVs using membrane blots. Protein membrane blots showing changes in mouse plasma cytokines before and after intravenous administration of PBS (control), 15 μg of pEVs, or 15 μg of *Δlgt-*pEVs in male or female mice. Three mice were pooled for each blot. Each row is a letter (from A to F), and each column a number (from 1 to 24). The coordinates are as follows. A1,A2,A23,A24,F1,F2: reference spots. F23,F24: negative control. B1,2: BLC, B3,4: C5/C5s, B5.6:G-CSF, B7,8: GM-CSF, B 9,10: I-309, B11,12: Eotaxin, B13,14: SICAM-1, B15,16: IFN-y, B17-18: IL-1α, B19-20: IL-1β, B21-22: IL-1ra, B23,24: IL-2, C1,2: IL-3, C3,4: IL-4, C5,6: IL-5, C7,8: IL-6, C9,10: IL-7, C11,12: IL-10, C13,14: IL-13, C15,16: IL12p70, C17,18: IL-16, C19,20: IL-17, C21,21: IL-23, C23,24: IL-27, D1,2: IP-10, D3,4: I-TAC, D5,6: KC, D7,8: M-CSF, D9,10: JE, D11,12: MCP-5, D13,14: MIG, D15,16: MIP-1α, D17,18: MIP-1β, D19,20:MIP-2, D21,22: RANTES, D23,24: SDF-1, E1,2 TARC, E3,4: TIMP-1, E5,6:TNF-α, E7,8: TREM-1. Download FIG S3, TIF file, 1.8 MB. Copyright © 2021 Yerneni et al. 2021 Yerneni et al. https://creativecommons.org/licenses/by/4.0/ This content is distributed under the terms of the Creative Commons Attribution 4.0 International license.

### Local application of pEVs promotes immune cell recruitment.

**FIG 7:**
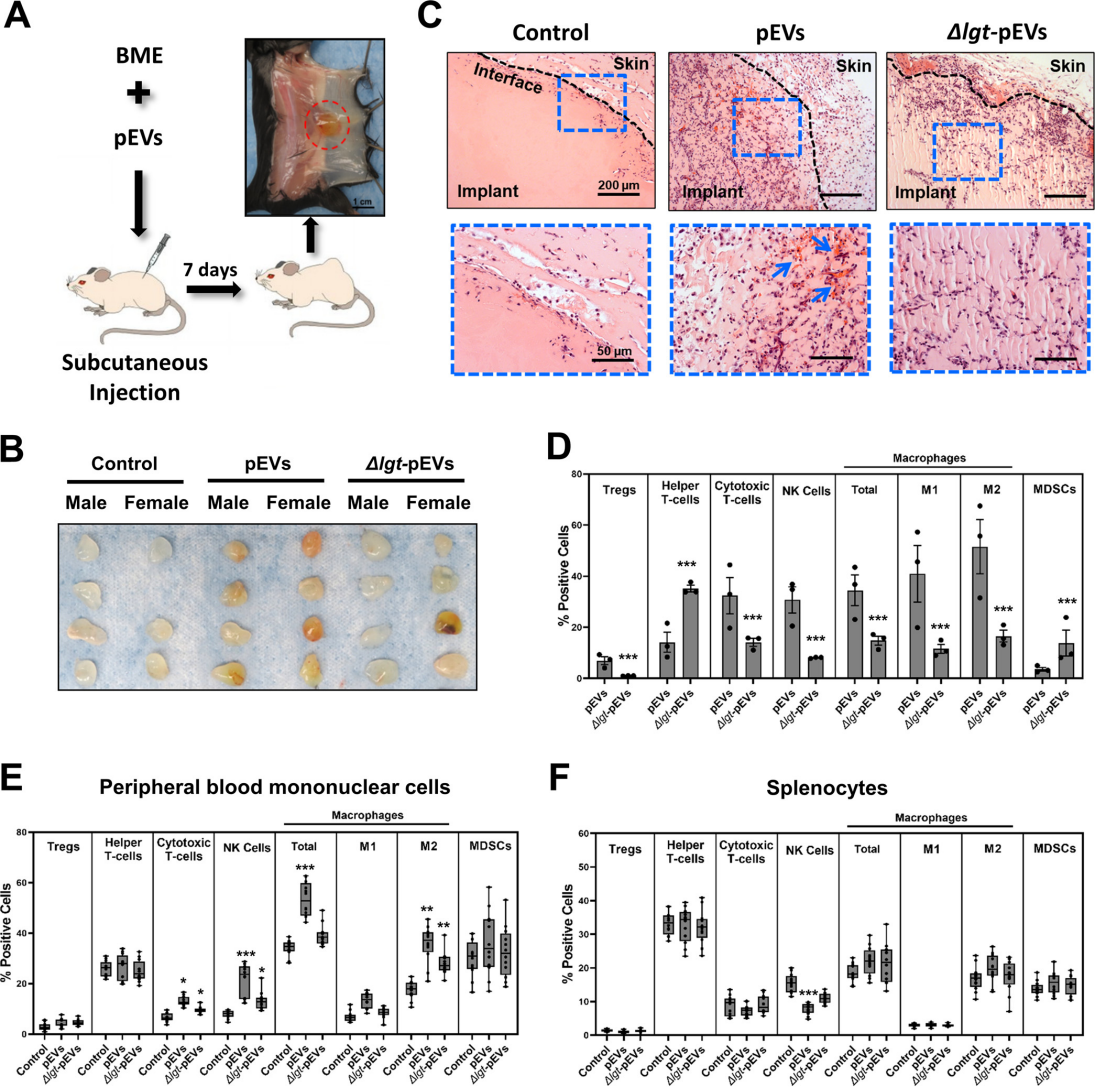
Local inoculation of pEVs in mice. Cohorts of 12 mice each, over two independent experiments, were injected with growth factor-reduced basement membrane extract hydrogel (0.5 ml) containing PBS control (0.1 ml), pEVs purified from wild-type bacteria (15 μg in 0.1 ml of pEVs), or *Δlgt-*pEVs purified from the *Δlgt* strain (15 μg in 0.1 ml of *Δlgt-*pEVs) and analyzed 7 days postinoculation. (A) Experimental design schematic, where the red circle highlights the hydrogel at the experimental endpoint. (B) Photograph of hydrogel plugs harvested from the first independent experiment. In one PBS-treated female, the hydrogel was no longer present at the site of injection, and this animal was removed from the analysis. (C) Representative images of hydrogel slices stained with H&E. Top panels show images at the interface between the skin and the plug; bottom panels show magnified zones from the regions denoted in the left panels. (D to F) Cell markers were used to assess levels of different cell types using flow cytometry. The cell types were defined as follows: regulatory T cells (CD4^+^ and FoxP3^+^), helper T cells (CD3^+^, CD45^+^, and CD4^+^), cytotoxic T cells (CD3^+^, CD45^+^, and CD8^+^), all macrophages (CD45^+^, CD11b+, and F480+), M1 macrophages (CD45+, CD11b+, F480+, CD86+, and CD80+), M2 macrophages (CD45+, CD11b+, F480+, and CD206+), natural killer cells (CD45+ and NKp46+), and myeloid-derived suppressor cells (CD45+, CD11b+, and Gr1+). (D) Relative quantification of infiltrated cells in pEV and Δlgt-pEV hydrogel implant. For each cohort, implants from 12 animal were pooled into three groups to get enough cells for flow cytometry analysis. Bars indicate means ± the SEM (n = 3). (E and F) Changes in the relative percentages of PBMCs and splenocytes pre- and postinjection of hydrogel containing PBS or pEVs or Δlgt-pEV. Box-and-whisker plots show median, range 25 to 75%, and min-max values (n = 12).

EVs from mammalian cells bind to extracellular matrix (ECM) proteins in the tissue microenvironment. As a result, when mammalian EVs are secreted, a portion is sequestered locally creating immobilized EV microenvironments (42). Pneumococcus forms biofilms on mucosal surfaces, such as the nasopharynx during colonization, the middle ear during otitis media, and the sinus during rhinosinusitis (10, 17–19). We suppose that analogous to the mammalian EVs, pEVs produced by the bacteria are likely to be sequestered in biofilms. Thus, we investigated whether pEVs influence innate immune responses when administered locally in a controlled fashion. To this end, we immobilized pEVs in growth factor reduced basement membrane extract (BME), an ECM derived from decellularized Engelbreth-Holm-Swarm sarcoma to enable local delivery in mice. BME is liquid at 4°C and solidifies upon raising the temperature to 37°C. pEVs and ECM were mixed on ice to generate a homogeneous mixture, and this mixture was injected subcutaneously into mice, where it immediately solidified providing a local immobilized pEV microenvironment (Fig. 7A). Mice were injected with PBS, pEVs isolated from wild-type strain, or pEVs isolated from the *Δlgt* strain. At 7 days postinoculation, ECM hydrogels, PBMCs, and spleens were analyzed for changes in morphology, cell recruitment, and cytokine responses.

The pEVs promoted immune cell recruitment to the ECM hydrogel. Macroscopic analysis revealed differences in the visual appearance of the hydrogels between the control and experimental groups. Hydrogel plugs from control animals displayed an opaque-white color, while those from animals treated with pEVs displayed an orange hue consistent with the infiltration of red blood cells (RBCs) (Fig. 7B). With one exception, hydrogel from mice treated with *Δlgt*-pEVs resembled the controls. Microscopic analysis of H&E staining of the hydrogels at the interface between the skin and the implants revealed recruitment of host cells in a pEV-dependent manner (Fig. 7C). An intermediate number of cells are present on ECM plugs infused with *Δlgt*-pEVs, suggesting that lipoproteins contribute to the ability of pEVs to recruit host cells (Fig. 7C, lower set of panels). To determine the nature of the cells in the infiltrate, cells in the hydrogel were analyzed with 12 surface markers and sorted by flow cytometry (Fig. 7D). In the sample with wild-type pEVs, the majority of cells corresponded to macrophages, NK cells, and CD8^+^ T cells. The number of cells recovered from the control hydrogels was too low for analysis, consistent with histology. Moreover, in contrast to wild-type pEVs, helper T cells, and myeloid-derived suppressor cells (MDSCs) corresponded to the majority of cells in mice treated with *Δlgt*-pEVs, suggesting that the impact of lipoproteins varies in a cell-dependent manner. Together, these data suggest that pEVs induce efficient recruitment of immune cells into the hydrogel.

The influence of the local administration of pEVs extended to PBMCs. Most notable was an increase in macrophages and NK cell levels after treatment with wild-type pEVs (Fig. 7E; see also Fig. S5). Further, bacterial lipoproteins contributed to this effect, since it was diminished with Δ*lgt*-pEVs (Fig. 7E). The effect of local administration was minor in the spleen, where we only observed a decrease in NK cells (Fig. 7F). Notably, in contrast to systemic administration, local delivery of pEVs did not trigger cytokine signaling in the plasma (see Fig. S4). We conclude that the local accumulation of pEVs leads to the recruitment of host immune cells, demonstrating that pEVs are pneumococcal immune modulators.

10.1128/mBio.01657-21.4 FIG S4 Detection of plasma cytokines in mice inoculated (locally) with pEVs. Protein membrane blots showing levels of mouse cytokines before and after local injection of basement membrane extract hydrogel implant containing PBS (control) or 15 μg of pEVs or 15 μg of *Δlgt-*pEVs in male and female mice. Three mice were pooled for each blot. There may be a mild increase in BLC in females treated with pEVs and *Δlgt-*pEVs. Each row is a letter (from A to F), and each column a number (from 1 to 24). The coordinates are as follows: A1,A2,A23,A24,F1,F2: reference spots. F23,F24: negative control. B1,2: BLC, B3,4: C5/C5s, B5.6:G-CSF, B7,8: GM-CSF, B 9,10: I-309, B11,12: Eotaxin, B13,14: SICAM-1, B15,16: IFN-y, B17-18: IL-1α, B19-20: IL-1β, B21-22: IL-1ra, B23,24: IL-2, C1,2: IL-3, C3,4: IL-4, C5,6: IL-5, C7,8: IL-6, C9,10: IL-7, C11,12: IL-10, C13,14: IL-13, C15,16: IL12p70, C17,18: IL-16, C19,20: IL-17, C21,21: IL-23, C23,24: IL-27, D1,2: IP-10, D3,4: I-TAC, D5,6: KC, D7,8: M-CSF, D9,10: JE, D11,12: MCP-5, D13,14: MIG, D15,16: MIP-1α, D17,18: MIP-1β, D19,20:MIP-2, D21,22: RANTES, D23,24: SDF-1, E1,2 TARC, E3,4: TIMP-1, E5,6:TNF-α, E7,8: TREM-1. Download FIG S4, TIF file, 1.7 MB. Copyright © 2021 Yerneni et al. 2021 Yerneni et al. https://creativecommons.org/licenses/by/4.0/ This content is distributed under the terms of the Creative Commons Attribution 4.0 International license.

10.1128/mBio.01657-21.5 FIG S5 Gating strategy for lymphocytes (helper T cells/cytotoxic T cells), macrophages (M1/M2 macrophages), natural killer cells, and myeloid-derived suppressor cells. Download FIG S5, TIF file, 2.1 MB. Copyright © 2021 Yerneni et al. 2021 Yerneni et al. https://creativecommons.org/licenses/by/4.0/ This content is distributed under the terms of the Creative Commons Attribution 4.0 International license.

### pEVs induce alternatively activated macrophages.

In general, macrophages produce diverse responses depending on the type of stimulation. Macrophages can be polarized to the classical activation pathway with proinflammatory cytokines (referred to as M1; LPS is a common inducer) or the alternative pathway activation with arginase-1 production (referred to as M2; IL-4 is a common inducer). The strongest effect of systemically administered pEVs on PBMCs was a 4.5-fold increase in M2 macrophages (Fig. 4A and B). Similarly, upon local administration, M2 macrophages were recruited to gel plugs with pEVs and increased in PBMCs (Fig. 7D and E). These data suggest that pEVs induce macrophage polarization, with markers associated with the M2 state. Thus, pEV may alter the balance between macrophages differentiating from a classical phenotype (M1 polarization) to an alternative phenotype (M2 polarization).

**FIG 8:**
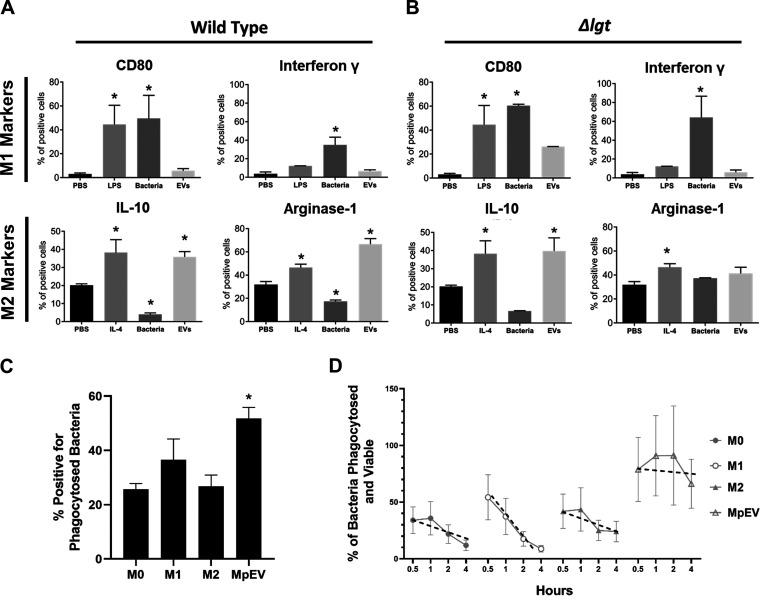
pEVs induce alternatively activated macrophages. Primary human macrophages were exposed to live bacteria (MOI = 5) or 20 μg/ml pEVs or 20 μg/ml *Δlgt-pEV.* To differentiate between the M1 macrophage phenotype and the M2 macrophage phenotype, cells were treated with 10 ng/ml LPS or 50 ng/ml IL-4, respectively, as controls. Graphs indicate the percent positive cells for the indicated cell markers that include classically activated “inflammatory” macrophages (IFN-γ and CD80 positive) or alternatively activated “immunoregulatory” macrophages (Arg-1 and IL-10 positive). (A) Response to wild-type bacteria and pEVs. (B) Response to *Δlgt* bacteria and *Δlgt-*pEVs. Bars represent means ± the SEM (*n* = 3). (C) Bar graph showing an assessment of bacteria phagocytosed by different subtypes of primary human macrophages. Bars indicate cells positive for phagocytosed bacteria after 1 h of infection with SYTO-9-labeled bacteria. Bars represent means ± the SEM (two independent experiments with *n* = 3). (D) Percentages of surviving bacterial cells (by viable plating) at different time points after internalization in macrophages. Points indicate means ± the SEM (*n* = 3). Black dotted line indicates the survival trend curve with slope representing the rate of bacterial death in macrophages.

To explore this hypothesis, we performed *in vitro* experiments with primary human macrophages to compare the cytokine response triggered by bacteria versus by pEVs. We used lipopolysaccharide (LPS) and interleukin-4 (IL-4) as controls for the classical and alternative pathways, respectively. Live pneumococci promoted an inflammatory response, with increases of the M1 markers gamma interferon (IFN-γ) and CD80 and a decrease of the M2 markers arginase-1 and IL-10 (Fig. 8A). In contrast, pEVs did not stimulate IFN-γ and CD80 and instead elevated levels of arginase-1 and IL-10, consistent with the alternative activation pathway (Fig. 8A). Moreover, bacterial lipoproteins contributed to the regulation of the alternative pathway, albeit the role of these proteins differs in the context of bacteria versus pEVs (Fig. 8B). Wild-type bacteria induced a decrease in arginase-1 levels, while pEVs induced an increased expression of arginase-1 (Fig. 8A). In contrast, *Δlgt* bacteria and *Δlgt*-pEVs did not influence the levels of arginase-1 (Fig. 8B). Thus, the nature of lipoproteins or their partners likely differ between bacterial cells and pEVs, leading to changes in the functional outcome. We conclude that pEVs are determinants of bacterial immunomodulation. Moreover, the influence of pEVs on macrophage signaling differs from that of bacteria. Bacteria promote the classic pathway, and pEVs promote activation of the alternative pathway in macrophages.

### Priming of macrophages with pEVs increases their phagocytic capacity and influences pneumococcal survival.

Macrophages play an important role in fighting pneumococcal infections by internalizing and killing the bacterial cells. Different subtypes of macrophages (M1/M2) are known to play different roles during infection and tissue regeneration (43). Therefore, we questioned whether pEV treatment activated human primary macrophages to have different phagocytic capacity compared to classical or alternatively activated macrophages. Pretreatment with pEVs increased the phagocytic capacity of human primary macrophages >2-fold compared to untreated (nonactivated) control macrophages (Fig. 8C). We extended this analysis to a model system, using J774A.1 murine macrophages, where we quantified both percent phagocytosis as well as viable bacteria over time. Murine macrophages were unprimed (M0), primed with LPS (M1), primed with IL-4 (M2), or primed with pEVs. The percent phagocytosis was measured 30 min after the addition of pneumococcus, and viable intracellular bacteria were measured at 1, 2, and 4 h postinternalization. The extent of phagocytosis was higher in macrophages primed with pEVs. However, once phagocytosed, bacteria on average survived longer in these macrophages relative to other conditions. The effect of pEVs on bacterial survival inside macrophages is variable across experiment, which may reflect the inherent heterogeneity of EVs. Nonetheless, these data together suggest that pEVs may reprogram macrophages to increase bacterial survival (Fig. 8D).

### pEV-associated pneumolysin contributes to macrophage phagocytosis and bacterial survival.

The pneumococcal toxin pneumolysin is commonly known for its pore-forming activity. However, it also influences host cells via binding to the host mannose receptor C type 1 (MRC1 [44]). This receptor binding inhibits TLR signaling and proinflammatory cytokines in murine macrophages and, in a murine model of pneumococcal disease, induction of MRC-1 is associated with increased bacterial loads. Pneumolysin is present on the surface of EVs (20), and MRC-1 is expressed in primary macrophages (see Fig. S8). Thus, we hypothesized that pneumolysin may mediate the polarization of macrophages toward the alternative M2 phenotype (45–47). Specifically, we hypothesized that the induction of phagocytosis and/or survival of bacteria in pEV-pretreated macrophages could be a function of pEV associated pneumolysin interaction with MRC-1.

10.1128/mBio.01657-21.8 FIG S8 Primary human macrophages express MRC-1 (CD206). The percent CD206-positive primary human macrophages after exposure to PBS or to IL-4 are shown. Download FIG S8, TIF file, 1.4 MB. Copyright © 2021 Yerneni et al. 2021 Yerneni et al. https://creativecommons.org/licenses/by/4.0/ This content is distributed under the terms of the Creative Commons Attribution 4.0 International license.

**FIG 9:**
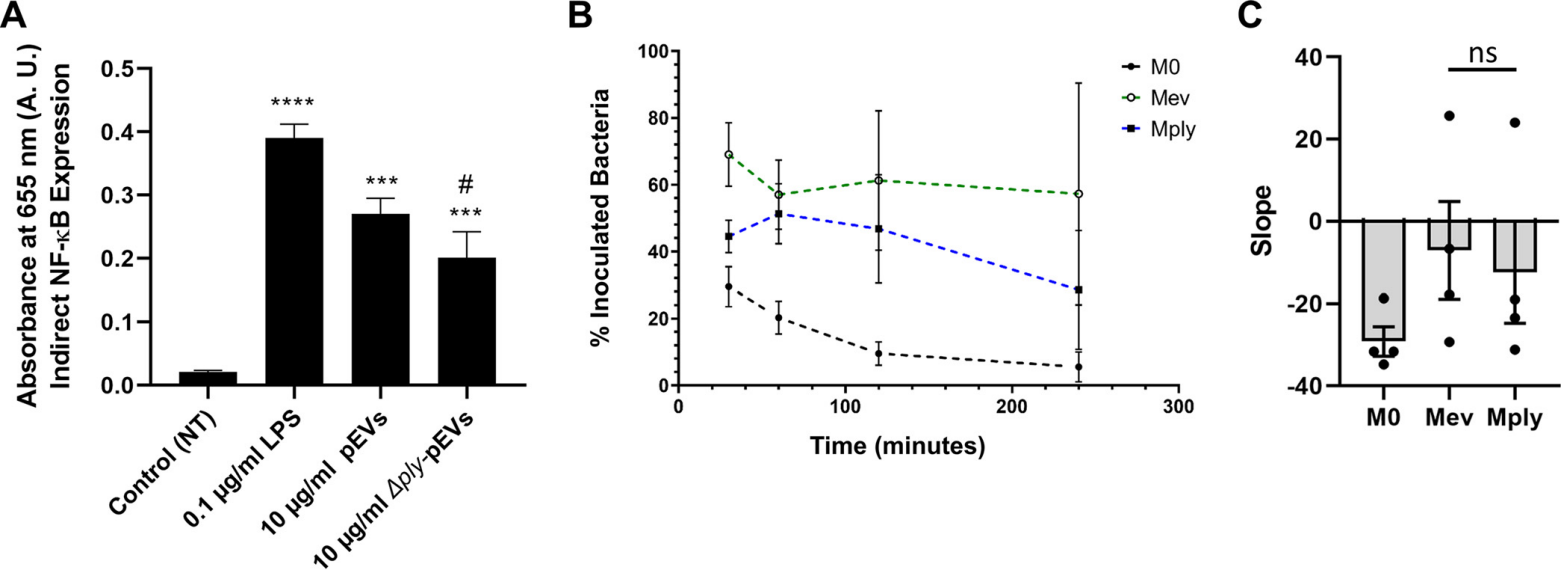
Pneumolysin contributes to pEV-induced macrophage activation and phagocytosis. (A) NF-κB production in murine macrophage reporter cell line (RAW-Blue) is reduced for *Δply-*pEVs compared to pEVs. Bars represent the means ± the SEM of three independent experiments (*n* = 3; all conditions run in parallel). ****, *P*  ≤ 0.0001; ***, *P*  ≤ 0.001 (versus control group); #, *P*  ≤ 0.01 (versus pEV group). (B) Percentages of surviving bacterial cells (by viable plating) at different time points after internalization in macrophages. Points indicate means ± the SEM (*n* = 4). The dotted black line indicates the survival trend curve, with the slope representing the rate of bacterial death in macrophages. (C) Bar graph showing slopes of bacteria survival plot shown in panel B. The percentages of viable and internalized bacteria over time were fitted to a linear curve. The slope of the fitted trend line was used as an estimate for rate of bacterial death. Slopes of individual experiments are depicted as dots. Bars represent means ± the SEM (*n* = 4).

We first tested whether pneumolysin contributes to pEV induction of NF-κB on macrophages (Fig. 3). We generated pEVs from a pneumolysin deletion mutant (Δ*ply*). Using the murine macrophage reporter cell line, we observed an intermediate phenotype for the *Δply*-pEVs. They significantly induced NF-κB, but to a lesser extent than pEVs from wild-type cells (Fig. 9A). Next, we tested the influence of pneumolysin on the ability of pEVs to increase phagocytosis. Pretreating macrophages with *Δply*-pEVs increased their phagocytic potential compared to untreated macrophages (M0), as indicated by the increased amount of intercellular pneumococci at 30 min postinoculation, but it remained lower than the macrophages that were pretreated with wild-type pEVs (Fig. 9B). We further measured the survival of internalized bacteria at 1, 2, and 4 h postexposure to macrophages. As shown previously, in M0 untreated macrophages, bacteria display a low survival rate with few surviving pneumococci by 2 h. In contrast, bacterial survival is significantly higher in cells pretreated with pEVs from wild-type cells, albeit with high variability across experiments (likely due to heterogeneity of vesicles) (Fig. 9B and C). Bacterial survival displayed an intermediate phenotype after treatment with *Δply*-pEVs compared to wild-type pEVs or no treatment. We conclude that pEVs modify macrophage responses via an increase in phagocytic capacity and decrease in induced bacterial death and that pneumolysin contributes to this response.

## DISCUSSION

Recent studies demonstrated that Gram-positive bacteria secrete EVs. However, questions remain about the mechanism(s) EVs utilize for trafficking through the thick cell wall of Gram-positive bacteria. Several hypotheses have been proposed. One hypothesis is that malleable EVs “squeeze” through openings in the cell wall. An alternative hypothesis proposes that pores in the cell wall facilitate trafficking of bacterial EVs (2). It has been also suggested that the release of EVs occurs whenever the bacterial cell wall is undergoing degradation, implying that Gram-positive EVs are a by-product of cell death instead of an active process (48, 49). Therefore, determining whether viable cells produce EVs is critical to experimental design and interpretation of pEVs biology in microorganisms with thick cell walls. Data countering the cell death hypothesis have been accumulating over the last decade (2, 18, 22). Moreover, experiments in the pneumococcus have demonstrated a direct association between EV production and live cells and showed that pEVs are not efficiently isolated from dead cells (22). Similarly, our experiments show that pEVs accumulate in cultures where the vast majority (∼95%) of cells are viable. When pneumococcus is grown in medium that inhibits autolysis, the pEV yield is not altered, further supporting pEV production by growing cells. Solving the puzzle of pEV biogenesis is a critical step in the field, and its solution will enable the manipulation of bacterial vesicle production opening several research avenues. The molecular mechanisms of Gram-positive EV biogenesis and trafficking through the cell wall remain unknown and are the subject of intense current research.

While the number of reports investigating pEVs remains limited, the yield and characteristics of pEVs isolated using different techniques remain conserved across studies. In this study, pEVs were purified by SEC from a mid-log-phase culture resulting in a protein yield of 45 ± 6.5 μg/ml (from a culture of 0.5 liter). These values are comparable to previous work where pEVs were purified by filtration and ultracentrifugation (20, 22). Regarding pEV size, we report a peak at 127 nm in diameter but with a broad size range (Fig. 1B). Nanoparticle tracking analyses (NTA) of EVs from all the different strains used in this study are shown in the Fig. S7. Codemo et al. (20) report EVs from 25 to 250 nm (with peaks between 25 and 75 nm), and Olaya-Abril et al. (22) report EVs between 20 and 80 nm in diameter. While isolation of pEVs across laboratories did not result in major differences, variations in purification and quantification methodologies may lead to differences in size estimates. Furthermore, characterization methodology can bias size estimates. We chose SEC to isolate pEVs since this technique is reported to be a gentle method of obtaining nonaggregated and partly “purified” EVs from mammalian sources (50).

10.1128/mBio.01657-21.7 FIG S7 Representative curves of NTA characterization of pEVs from wild-type, *Δlgt*, and *Δply* strains, selected from at least two independent profiles. Download FIG S7, TIF file, 1.8 MB. Copyright © 2021 Yerneni et al. 2021 Yerneni et al. https://creativecommons.org/licenses/by/4.0/ This content is distributed under the terms of the Creative Commons Attribution 4.0 International license.

Another major challenge in bacterial EV biology is to determine physiologically relevant concentrations of pEVs in diverse tissues and stages of infection. In planktonic cultures, we measure ∼5 × 10^9^ vesicles per 1 × 10^11^ cells. Estimates of the number of cells in an *in vivo* infection are challenging and predicted to vary dramatically depending on strain, microbiome, host immunity, tissue, and duration of chronic infection. There is also no evidence to suggest that pEV production rates, or even pEV cargo compositions, are the same between cells grown in planktonic or biofilm modes of growth. In support, quantitative and qualitative differences were observed from EVs produced by planktonic and biofilm cultures of P. aeruginosa (51). Thus, the various pEV studies draw the conclusion that the influence of pEVs on disease outcomes may be influenced by differences in the dose and composition of pEVs between experiments and infections.

EVs secreted by pathogens have been touted as potential vaccine candidates (49). For clinical translation of such vaccines a thorough understanding of interaction between the pathogen-derived EVs and host immune system *in vivo* is critical. Our study is the first in-depth study of *in vivo* effects of pEVs. Systemic injection of pEVs in mice led to a dramatic increase of cytokines in the plasma, as well as an increase in circulating macrophages and decrease in circulating helper T cells and NK cells. Interestingly, this reduction in helper T cells and NK cells was dependent on pEV-associated lipoproteins. Given that helper T cells are important players in stimulating the adaptive immune system, our data suggest that *Δlgt*-pEVs could potentially serve as a better systemically administered vaccine candidate over wild-type pEVs. Recent development in vaccine delivery strategies have also shown that a localized biomaterial-based strategy could offer the advantage of minimizing undue side effects that could arise from systemic delivery of vaccines (52). Given the substantial increase in cytokines observed in mice upon systemic delivery of pEVs, we studied whether hydrogel-based localized delivery of immobilized pEVs could offer an alternate approach to deliver pEV-based vaccines. Interestingly, the cells recruited into the pEV hydrogel contained a mix of immunostimulatory and immunoregulatory immune cells, suggesting that a more detailed study is necessary to assess the potential of localized delivery of pEVs for vaccination.

In the context of an infection, a single bacterial cell may lead to sepsis (53, 54). It is currently not fully understood how pneumococcal septicemia is initiated by one or more cells and how the bacterial cells survive the host immune system. Recent studies have shown that CD169^+^ splenic macrophages can act as reservoirs assisting pneumococcal cells to replicate and increase in number inside the host (55). Could pEVs be involved in assisting host immune evasion and bacterial replication and, in doing so, serve to promote sepsis? Our data indicate that pEV-priming increases the recruitment and the phagocytic capacity of macrophages and may also promote the survival of internalized pneumococci. Moreover, pneumolysin contributes to these processes. Thus, it is tempting to hypothesize that pEVs that migrate from the site of infection may condition macrophages to act as reservoirs, providing a survival benefit to bacteria in immunocompetent hosts.

This study was centered on pEVs produced by a nonencapsulated strain. The pEVs display reduced capsule components (22); thus, the composition of pEVs from nonencapsulated bacteria likely resembles those of their capsulated counterparts. In addition, the rate of pEV production is likely to be lower for encapsulated cells. A study comparing pEV production between the nonencapsulated R6 and encapsulated strains ST1, ST6B, ST8, and ST23F determined higher yields in R6 (22). However, we have not encountered a direct comparison between strains of the same genetic background that vary only by capsule type, so genetic differences between strain and their influence on efficiency of pEV production may be important. In this context, the timing of pEVs production during infection is likely to influence both composition and efficiency, since adherent cells downregulate production of the capsule. Further, cargo selection may lead to changes in the relative ratio of membrane proteins between whole cells and pEVs, which, in turn, may influence their function. For example, our findings suggest that lipoproteins are critical for manipulation of arginase-1 in macrophages (Fig. 8A and B) since *Δlgt* cells and *Δlgt*-pEVs have little to no effect on the percentages of macrophages expressing arginase-1. However, stimulation with bacteria decreases this marker, whereas stimulation with pEVs leads to increases. These differences in function may depend on differences in the context of lipoprotein presentation between cells and the pEVs they produce. In eukaryotic systems, especially in the context of tumor-derived EVs, mounting evidence suggests that the molecular profile of EVs could potentially carry cargo with unique isomeric forms compared to the profile of EV-secreting cells (56). For example, Ko et al. recently identified a unique isoform of vascular endothelial growth factor that is potentially present only on EVs secreted by tumor cells (57). This suggest that pEVs could potentially carry unique isoforms of lipoproteins with different bioactivity compared to the isoforms present on the EV-secreting pneumococcal cells.

The ability of pEVs to activate the alternative pathway (M2 polarization) suggests that pEVs promote pneumococcal chronicity. Chronic colonization of the upper respiratory tract is a prerequisite for disease and a critical step in the lifestyle of this exclusive human colonizer. Colonization of the nasopharynx is asymptomatic, and disease develops when the bacteria spreads to other tissues. IL-10 is a marker of the alternative pathway. Previous work on pneumococcal EVs has observed that they induce IL-10 production in dendritic cells (32, 58). Relative to wild-type mice, those with a knockdown of IL-10 display elevated levels of proinflammatory cytokines, increased recruitment of neutrophils into tissues, and lower bacterial loads (59). However, these knockout mice also display increased mortality, suggesting that IL-10-mediated control of the immune system benefits the host by fostering chronicity over dissemination and benefits the bacteria by promoting long-term carriage (59).

We conclude that pEVs fit into the category of pneumococcal immune modulators. They manipulate the adaptive immune response by influencing recruitment of immune cells and cytokine production. Our data suggest that upon pneumococcal infection pEVs are internalized into host cells. These pEVs promote host defense by triggering immune cell recruitment and cytokine responses. On the other hand, these pEVs also promote pneumococcal chronicity by providing an anti-inflammatory environment for bacterial survival. Thus, we propose that pEVs are pivotal effectors in the delicate interplay between bacteria and host, which determines whether the outcome of infections is clearance, carriage, or dissemination.

## MATERIALS AND METHODS

### Bacterial strains and growth conditions.

The primary strains used here are Streptococcus pneumoniae R6 and mutants created in this background. Bacterial strains were grown from frozen stocks by streaking on tryptic soy agar (TSA) plates containing 5% sheep blood, followed by incubation overnight at 37°C with 5% CO_2_. Liquid cultures were grown by picking colonies from TSA plates and inoculating them into Columbia broth. Liquid cultures are grown at 37°C with 5% CO_2_ without shaking.

### Generation of *lgt* deletion mutant strain.

The *lgt* and *ply* gene was deleted by insertion of a spectinomycin resistance cassette in its place by homologous recombination. This was achieved by PCR amplification of 2,000-bp regions immediately upstream and downstream of the *lgt* or *ply* gene that were joined through Gibson assembly to a spectinomycin resistance cassette to form a continuous linear DNA fragment (primers listed in Table S1 in the supplemental material). This DNA fragment was then transformed into S. pneumoniae strain R6 by natural transformation. The transformation was done by growing a culture in Columbia broth to an optical density of 0.05 and then mixing 1 ml of culture with the entire Gibson assembly reaction and CSP1 (0.125 μg/ml final concentration). This mixture was incubated at 37°C for 2 h and then plated on Columbia agar plates with spectinomycin (200 μg/ml) at 37°C overnight. Colonies were picked and confirmed by PCR and sequencing.

10.1128/mBio.01657-21.9 TABLE S1 Primers designed for pneumococcal strain R6. Download Table S1, TIF file, 2.0 MB. Copyright © 2021 Yerneni et al. 2021 Yerneni et al. https://creativecommons.org/licenses/by/4.0/ This content is distributed under the terms of the Creative Commons Attribution 4.0 International license.

### Mammalian cell culture. (i) Cell lines.

Mouse J774A.1 cells (TIB-67; ATCC, Manassas, VA) were grown and maintained in Roswell Park Memorial Institute medium (RPMI; Gibco, Gaithersburg, MD) supplemented with 10% heat-inactivated fetal bovine serum (HI-FBS; Invitrogen, Carlsbad, CA) and 1% penicillin-streptomycin (PS; Invitrogen). J774A.1 cells were certified by IDEXX BioAnalytics (Columbia, MO) to be free of bacterial, fungal, or mycoplasmal contamination. Human A549 cells (CCL-185; ATCC, Manassas, VA) were grown in Dulbecco modified Eagle medium (Invitrogen) containing 10% FBS and 1% PS.

### (ii) Primary T cell culture.

Blood samples were obtained from healthy donors at the University of Pittsburgh, Pittsburgh, PA (UPCI 09-069/IRB991206). PBMCs were isolated according to standard protocols and as previously described in detail (60). Briefly, blood samples from healthy volunteers (30 to 40 ml) were drawn into heparinized tubes and centrifuged on Ficoll-Hypaque gradients (GE Healthcare Bioscience, Chicago, IL). PBMCs were recovered, washed in AIM-V medium (Invitrogen), and immediately used for experiments. Naive CD4^+^ and CD8^+^ T cells were magnetically isolated using CD4^+^/CD8^+^ microbeads (Miltenyi, San Diego, CA) and used for subsequent experiments.

### (iii) Primary macrophage culture.

Primary human macrophages were isolated as previously described (61). Briefly, 7.5 × 10^6^ PBMCs (10% monocytes) were seeded in 6-well plates. After 2 h of incubation, cells were washed gently five times with PBS (pH 7.4) to remove nonadherent cells, and adherent cells were considered monocytes. For differentiation of PBMCs into macrophages, 2 ml/well of the growth medium RPMI 1640 supplemented with 10% (vol/vol) HI-FBS and 50 ng/ml GM-CSF (granulocyte-macrophage colony-stimulating facto, catalog no. 300-3; PeproTech, Rocky Hill, NJ) was used. Media were renewed after 4 days, and differentiation was completed after 7 days, after which the media were used for subsequent experiments.

### Isolation of pEVs.

pEVs were isolated at mid to late exponential phase according to previous studies which demonstrated high yields of pEVs at this point of the growth phase (22). pEVs from pneumococcal culture were isolated by SEC according to a previously described protocol (62). Briefly, conditioned medium was centrifuged at 10,000 × *g* for 20 min at 4°C. The supernatant was passed through a 0.22-μm Millipore filter. The filtered supernatant was concentrated 500-fold using 100-kDa molecular-weight cutoff filters (Thermo Scientific Pierce, Rockford, IL) that were centrifuged at 2,500 × *g* at 4°C until appropriately concentrated. pEVs were isolated from the concentrated supernatant by mini-SEC using minicolumns (1.5 × 12 cm; Econo-Pac columns; Bio-Rad, Hercules, CA) packed with 10 ml of Sepharose CL-2B (Sigma-Aldrich, St. Louis, MO) equilibrated with PBS. The supernatant (1.0 ml) was loaded onto the column, and five 1-ml fractions corresponding to the void volume peak were collected by running PBS over the column. Fraction 4 was used for subsequent experiments. Isolated pEVs were either used immediately (within 24 h) for subsequent experiments or stored at –80°C for long-term storage.

### Characterization of pEVs. (i) Protein content.

Protein concentrations of pEVs were determined using a BCA protein assay (Pierce Biotechnology, Rockford, IL) according to the manufacturer’s instructions.

### (ii) Nanoparticle tracking analysis.

Vesicles present in purified or unpurified samples were analyzed by nanoparticle tracking as previously described (42, 63), using the NanoSight LM10 system (NanoSight, Ltd., Amesbury, UK), configured with a 405-nm laser and a high-sensitivity digital camera system (OrcaFlash2.8, Hamamatsu C11440; NanoSight, Ltd.). The camera shutter speed was fixed at 30.01 ms, and camera gain was set to 500. Videos were collected and analyzed using the NTA software (version 2.3), with the minimal expected particle size, minimum track length, and blur setting all set to automatic.

### (iii) Transmission electron microscopy.

Transmission electron microscopy characterization was performed as previously described (64). Briefly, isolated total pEVs were fixed with 4% glutaraldehyde (Electron Microscopy Services, Hatfield, PA) for 20 min at room temperature. A 10-μl droplet of glutaraldehyde-fixed pEVs was placed on Formvar-coated 300 mesh copper grid (Electron Microscopy Services). The sample was incubated for 1 min, followed by rinsing with distilled water for 1 min to ensure the removal of PBS salts. Excess liquid was blotted off with a Whatman filter. After rinsing, 50 μl of uranyl acetate solution was put on the grid and allowed to remain for 1 min. Excess liquid was removed, and the grids were viewed on a Hitachi H-7100 transmission electron microscope (Hitachi High Technologies) operating at 100 keV. Digital images were collected using an AMT Advantage 10 CCD camera system (Advanced Microscopy Techniques) and inspected using NIH ImageJ software (http://rsbweb.nih.gov/ij/).

### pEV internalization studies. (i) Confocal microscopy.

pEVs were labeled with Vybrant-DiD (Invitrogen) according to the manufacturer’s instructions and as previously described (65, 66). DiD-labeled pEVs were incubated with J774A.1 and A549 cells for designated time points. Internalization experiment was performed in a 48-well plate so the volume in each well was 250 μl, and the pEVs added were to a total concentration of 20 μg/ml. pEVs were concentrated to 30 μg/100 μl after isolation before adding them into each well. To remove plasma membrane-bound pEVs, cells were treated with stripping buffer (500 μM NaCl and 0.5% acetic acid in deionized water [pH 3]) for 45 s, followed by three washes with PBS. Cells were fixed with 3.33% freshly prepared paraformaldehyde (PFA; Electron Microscopy Services) for 20 min at room temperature. Excess fixative was quenched by adding an equal volume of 1% (wt/wt) bovine serum albumin (BSA) in PBS for 5 min, followed by three washes with PBS. Fixed cells were permeabilized with 0.1% Triton X-100 in PBS for 1 min. To visualize F-actin and nuclei, cells were stained with Alexa Fluor 488-phallodin (5:200 in PBS; Thermo Fisher Scientific, Waltham, MA) and Hoechst 33342 (1:1,000 in PBS; Thermo Fisher Scientific), respectively. Imaging was performed using a Carl Zeiss LSM 880 confocal microscope with fixed settings across all of the experimental time points, and the images were analyzed using ZEN Black software (Carl Zeiss Microscopy, Thornwood, NY). The data are from two independent experiments, each one performed in triplicate.

### (ii) Flow cytometry tracking.

Portions (10 μg/ml) of DiD-labeled pEVs were added to J774A.1/A549/CD4^+^/CD8^+^ cells for the indicated times and then analyzed for red fluorescence posttreatment with stripping buffer. Flow cytometric analysis was performed on an Accuri C6 flow cytometer (BD Biosciences, San Jose, CA) connected to an Intellicyt HyperCyt autosampler (IntelliCyt Corp., Albuquerque, NM) using the red (649-nm) channel. Data were processed and interpreted using FlowJo software (FlowJo LLC, Ashland, OR).

### NF-κB reporter assay.

RAW-Blue cells (murine RAW 264.7 macrophage reporter cell line) were purchased from InvivoGen (San Diego, CA). This reporter cell line stably expresses a secreted embryonic alkaline phosphatase (SEAP) gene inducible by NF-κB activation that can be detected based on calorimetry. The assay was performed according to manufacturer’s instructions. Briefly, 20,000 RAW-Blue cells were treated with indicated treatments in 96-well plates and incubated for 24 h under culture conditions (37°C, 5% CO_2_, and 95% relative humidity). Postincubation, 20 μl of conditioned medium was collected, followed by incubation with 200 μl of QUANTI-blue reagent (InvivoGen) and optical density at 655 nm was measured using a Tecan spectrophotometer (Tecan, Männedorf, Switzerland). The data are from two independent experiments, each one performed in triplicate.

### Immunostaining.

Macrophages (2 × 10^5^ cells) were seeded on rat tail collagen type 1-coated coverslips. After incubation of 12 h, which was performed for attachment, cells were treated as indicated for 30 min. After the treatments, the cells were fixed in 3.33% paraformaldehyde or 20 min at room temperature. After four washes with PBS, cells were permeabilized with 0.1% Triton X for 10 min at room temperature. The cells were then washed with three washes of PBS, followed by incubation with p65 antibody (1:100 in PBS; ab16502; Abcam, Cambridge, UK) overnight at 4°C, and then washed and incubated with a secondary antibody (1:500 in PBS; mouse anti-rabbit Alexa Fluor 488; Invitrogen) for 2 h at room temperature. The cells were then incubated with Hoechst (1:1,000 in PBS; Invitrogen) for 5 min and mounted using Prolong gold solution (Thermo Fisher Scientific). Imaging was performed with a Carl Zeiss LSM 880 confocal microscope at fixed settings across treatments.

### Macrophage phenotype studies.

After differentiation, macrophages (up to 95% of the cells) were maintained in RPMI 1640 supplemented with 10% (vol/vol) HI-FBS for 24 h. Cells were treated either with PBS as a control, with LPS (100 ng/ml; Sigma-Aldrich) to induce M1 polarization, or with IL-4 (50 ng/ml; R&D Systems) to induce M2 polarization. pEVs (10 μg/ml) from wild-type or *Δlgt* bacteria were concentrated and added to the medium of macrophages, or else macrophages were directly cocultured with bacterial cells with a multiplicity of infection (MOI) of 5.

After 24 h of incubation, the phenotype was assessed by flow cytometry. For intracellular staining, cells were washed with PBS and fixed with eBioscience IC fixation buffer (catalog no. 00-8222-49) for 20 min at room temperature. Cells were then permeabilized with eBioscience permeabilization buffer (catalog no. 00-8333-56). For surface or intracellular staining, the cells were coincubated with anti-CD80-PE (1:3, 557227; BD Biosciences), anti-CD86-PE (1:3, PNIM2729U; IO Test), anti-HLA-DR-FITC (1:3, PNIMO436U; IO Test), anti-EGFR-APC (1:10, 352906; BioLegend), anti-CD206-FITC (1:3, 551135; BD Biosciences), anti-IL-10-PE (1:3, 559330; BD Biosciences), anti-LAP-PE (1:3, 349604; BioLegend), anti-Arginase-1-FITC (1:5, IC5868F; R&D Systems), anti-CD39-FITC (1:25, 328206; BioLegend), or anti-CD73-FITC (1:25, 344016; BioLegend) and with appropriate isotype controls in staining buffer (PBS + 3% BSA) for 30 min at room temperature with a minimum of two washes with PBS after each incubation. Cell fluorescence was measured in 10,000 events with an Accuri flow cytometer according to the manufacturer’s instructions (BD Biosciences).

### *In vivo* studies.

Animal care and experimental procedures were carried out at Carnegie Mellon University (Pittsburgh, PA) in accordance with the NIH *Guide for the Care and Use of Laboratory Animals* under an approved Institutional Animal Care and Use Committee protocol. All *in vivo* studies utilized C57BL/6 mice (6 to 8 weeks old, 22 to 28 g).

### (i) Systemic model.

Mice were injected intravenously with 15 μg of pEVs in 100 μl of PBS (stock solution of 1.5 mg/ml of pEVs) through the retro-orbital injection and, as a vehicle control, 100 μl of PBS was used. Prior to injection, ∼500 μl of blood was collected via submandibular bleeding and heparinized to avoid clotting. After 24 h, ∼500 μl of blood was again collected, and mice were euthanized.

### (ii) Local model.

Growth-factor-depleted basement membrane extract (400 μl; Culturex Trevigen, Gaithersburg, MD) was mixed with 100 μl of PBS (negative control) or 15 μg of total pEV protein in a volume of 100 μl, followed by incubation for 24 h on ice. Next, 100 μl of pEV stock solution (1.5 mg/ml) was mixed with 400 μl of hydrogel prior to injection. Mice were anesthetized, and the skin overlying the area of injection was gently shaved. Each mouse received two subcutaneous injections (negative control and pEVs) in the midventral abdominal region, which were permitted to solidify. Plugs were harvested after 7 days.

### Histology.

Mouse organs and hydrogel plugs were harvested and fixed in 10% buffered formalin (Sigma-Aldrich) for 24 h prior to embedding in paraffin blocks for histological analysis. The paraffin embedded specimens were sectioned at a thickness of 5 μm and stained with H&E.

### Cytokine arrays.

The relative levels of cytokines in mouse plasma were detected by using a proteome profiler mouse cytokine array kit (ARY006; R&D Systems, Inc., Minneapolis, MN). Aliquots of plasma (500 μl) were added to the array, and detection was performed according to the manufacturer’s protocol. The results were analyzed with the ImageJ software.

### *In vivo* flow cytometry.

Animals were sacrificed, and blood samples and their spleens were harvested. Spleens were mechanically dissociated and subsequently gently forced through a 70-μm strainer, followed by rinsing with 50 ml of PBS. Cells were pelleted by centrifugation (500 × *g* for 10 min at 4°C) and immediately resuspended in red blood cell RBC lysis buffer. RBC lysis was quenched by adding 3 volumes of PBS + 10% FBS, and cells were centrifuged at 500 × *g* for 10 min at 4°C. The cell pellet was resuspended in fluorochrome-conjugated antibodies: CD45-PE-Cy7 (1:50, 25-0451-82; Invitrogen), CD4-PerCP-Cyanine5.5 (1:50, 45-0042-80; Invitrogen), CD8-645 (1:50, 64-0081-80; Invitrogen), CD11b-Alexa Fluor 647 Cells (1:50, 557686; BD Biosciences), F4/80-eFluor450 (1:50, 48-4801-80; Invitrogen), CD80-BV510 (1:50, 740130; BD Biosciences), CD86-BV786 (1:50, 740877; BD Biosciences), CD206-PerCP/Cy5.5 (1:50, 141716, BioLegend), CD3-APC (1:50, 47-0031-80; Invitrogen), NKp46-FITC (1:50, 560756; BD Biosciences), and Gr-1-Alexa Fluor 700 (1:50, 56-5931-80; Invitrogen). Cells were stained with dye-conjugated antibodies for 60 min at room temperature in the dark, washed, fixed with IC fixation buffer (eBioscience), and stored at 4°C until analysis. Samples were analyzed on an LSR II flow cytometer with FACSDiva software (BD Biosciences). Compensation and analysis were performed using FlowJo software, and the gating strategy is shown in the Fig. S5 in the supplemental material.

### Phagocytosis assay.

Human primary macrophages were isolated as described above, and ∼1 × 10^5^ macrophages were seeded per well in a tissue culture plate and pretreated for 24 h as indicated (PBS/LPS/IL-4/pEVs). Cells were then infected with 25 μl of SYTO-9-labeled pneumococcal cells harvested at mid-log phase at an MOI of 5. After 1 h of infection, the cells were washed with PBS three times, treated with stripping buffer for 45 s, washed again with PBS three times, and finally trypsinized to detach them from plates. The extent of phagocytosis was determined using the green channel (488 nm) of an Accuri C6 flow cytometer (BD Biosciences). Data were analyzed using FlowJo software. The gating strategy is shown in Fig. S6.

10.1128/mBio.01657-21.6 FIG S6 Representative gating strategy for macrophages to assess phagocytosis. Priming of macrophages involved treating cells with 10 μg/ml pEVs for 24 h prior to inoculating them with bacteria. Download FIG S6, TIF file, 1.9 MB. Copyright © 2021 Yerneni et al. 2021 Yerneni et al. https://creativecommons.org/licenses/by/4.0/ This content is distributed under the terms of the Creative Commons Attribution 4.0 International license.

### Phagocytosis bacterial survival assay.

To test the phagocytosis of wild type bacteria and the different pneumococci mutants, 1 × 10^5^ J774A.1 cells were seeded per well in a tissue culture coated 12-well or 24-well plate (Corning, Inc., Corning, NY) and allowed to adhere overnight. Cells were pretreated for 24 h with either PBS, LPS, IL-4, or pEVs (total of 20 μg/ml in 1 ml per 400-μl or 200-μl volume). The isolated pEVs were first concentrated to ∼60 μg/ml using 100,000 MWCO filters (Vivaspin 500 concentrators; Sartorius AG, Germany) prior to adding them to macrophages. Thereafter, cells were infected with 25 μl of pneumococcal suspension containing ∼5 × 10^5^ CFU, resulting in an MOI of 5, followed by incubation at 37°C for 30 min. Postincubation, the cells were washed three times with PBS and then incubated for either 30, 60, 120, or 240 min in RPMI culture medium containing 10 μg/ml penicillin and 200 μg/ml gentamicin to kill extracellular pneumococci. The cells were then washed five times with PBS, and phagocytosed pneumococci were harvested by treating the infected J774A.1 cells with 0.025% saponin for 15 min at 37°C. Recovered bacteria were enumerated by viable plating on blood agar plates.

10.1128/mBio.01657-21.10 VIDEO S1 Confocal Z-stack video showing pEVs internalized by A549 cells. Download Movie S1, AVI file, 6.4 MB. Copyright © 2021 Yerneni et al. 2021 Yerneni et al. https://creativecommons.org/licenses/by/4.0/ This content is distributed under the terms of the Creative Commons Attribution 4.0 International license.
