# Supplementary figures and images for: Pneumococcal Extracellular Vesicles Modulate Host Immunity
Source: mBio. 2021 Jul 13;12(4):e01657-21. doi: 10.1128/mBio.01657-21 (PMC8406339; doi:10.1128/mBio.01657-21)

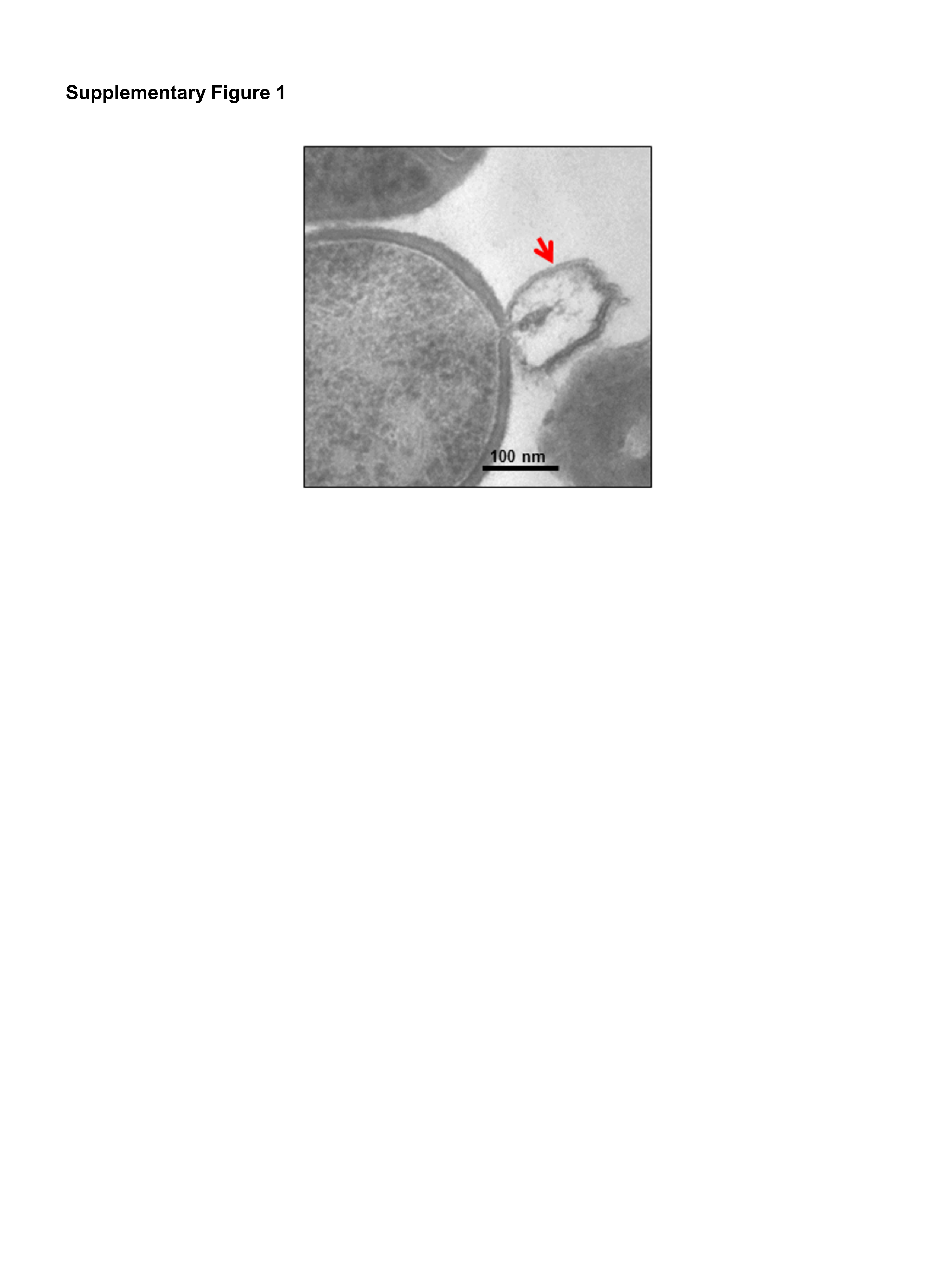

Supplement: FIG S1 [file mbio.01657-21-sf001.tif]

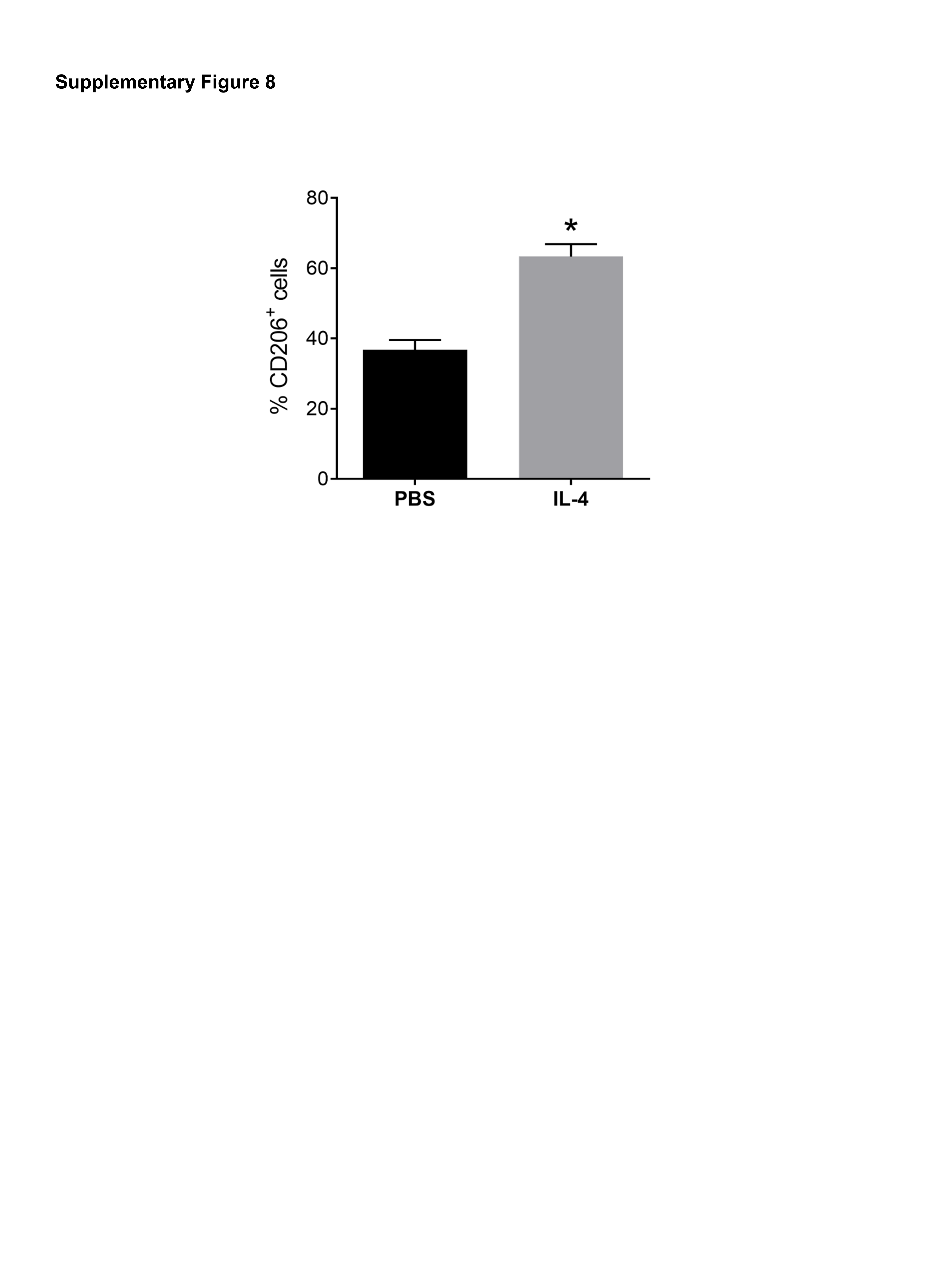

Supplement: FIG S8 [file mbio.01657-21-sf008.tif]

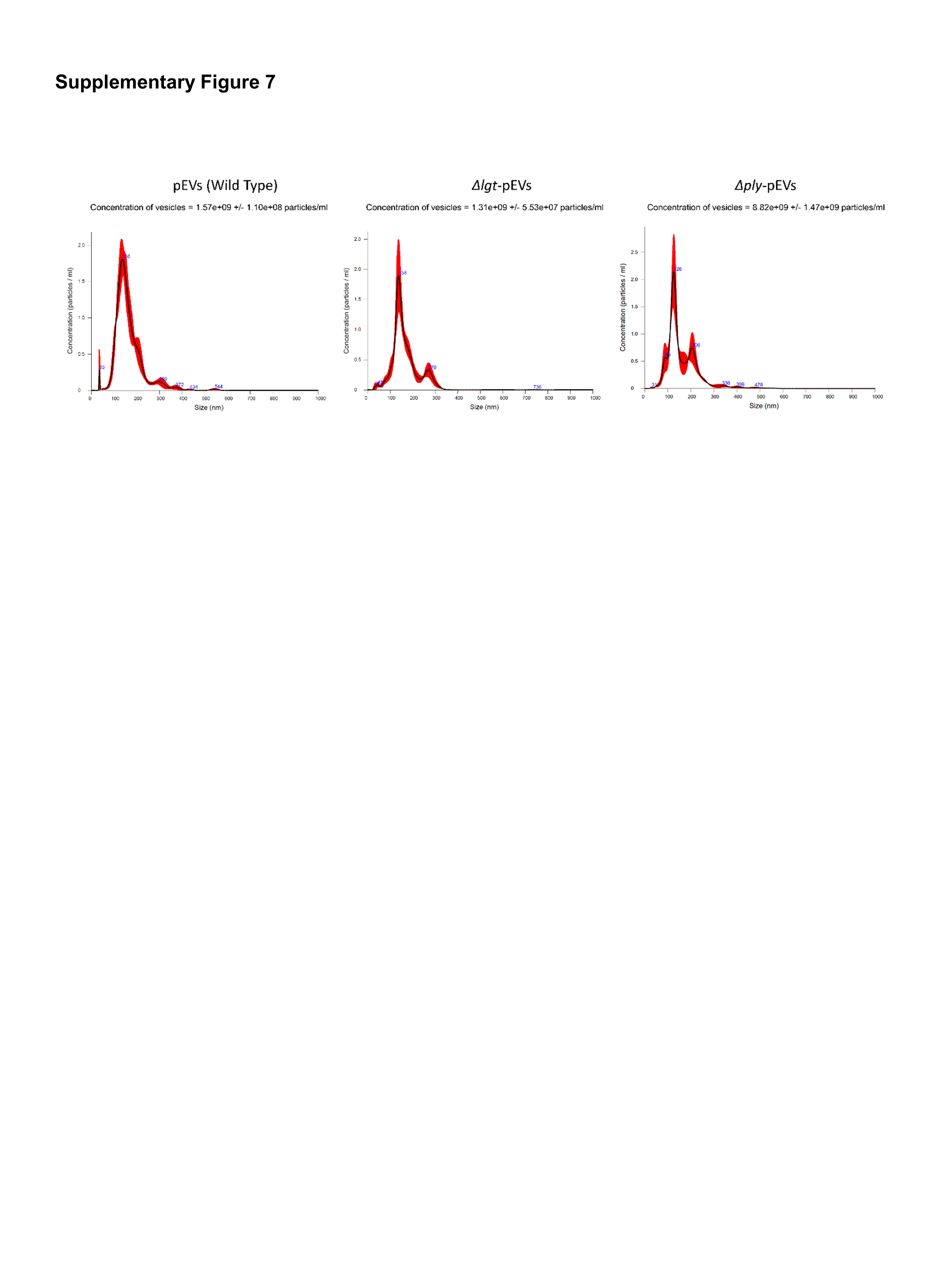

Supplement: FIG S7 [file mbio.01657-21-sf007.tif]

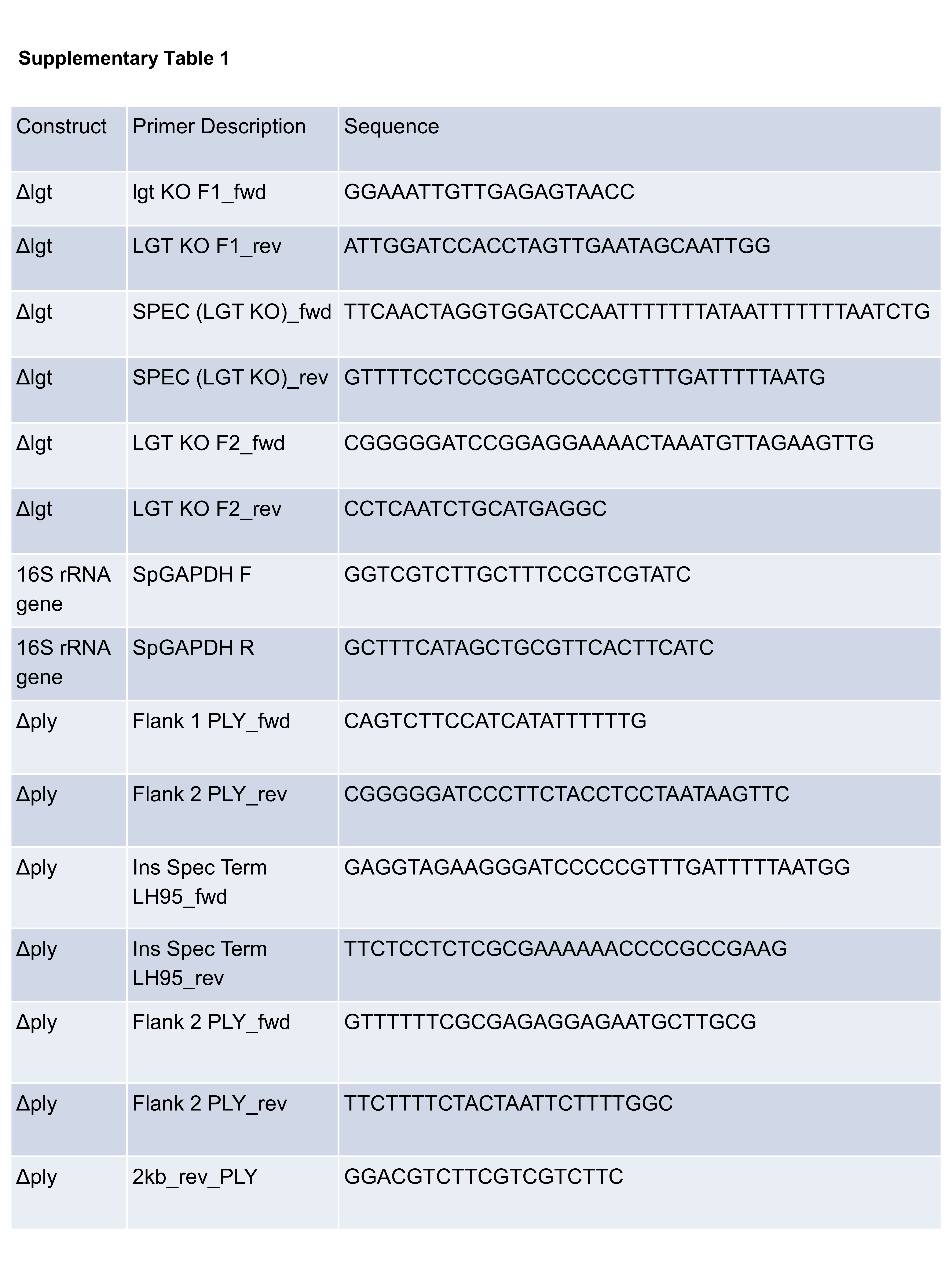

Supplement: TABLE S1 [file mbio.01657-21-st001.tif]
